# CST1 inhibits ferroptosis and promotes gastric cancer metastasis by regulating GPX4 protein stability via OTUB1

**DOI:** 10.1038/s41388-022-02537-x

**Published:** 2022-11-12

**Authors:** Dongbao Li, Yuhong Wang, Chao Dong, Tao Chen, Anqi Dong, Jiayu Ren, Weikang Li, Gege Shu, Jiaoyang Yang, Wenhao Shen, Lei Qin, Lin Hu, Jin Zhou

**Affiliations:** 1 Department of General Surgery, The First Affiliated Hospital of Soochow University, Suzhou, 215006 Jiangsu China; 2 Department of Pathology, The First Affiliated Hospital of Soochow University, Suzhou, 215006 Jiangsu China; 3 State Key Laboratory of Radiation Medicine and Protection, School of Radiation Medicine and Protection and School for Radiological and Interdisciplinary Sciences (RADX), Collaborative Innovation Center of Radiation Medicine of Jiangsu Higher Education Institutions, Soochow University, Suzhou, 215123 Jiangsu China

**Keywords:** Gastric cancer, Prognostic markers

## Abstract

Metastasis is an important factor contributing to poor prognosis in patients with gastric cancer; yet, the molecular mechanism leading to this cell behavior is still not well understood. In this study, we explored the role of cysteine protease inhibitor SN (Cystatin SN, CST1) in promoting gastric cancer metastasis. We hypothesized that CST1 could regulate gastric cancer progression by regulating GPX4 and ferroptosis. Whole transcriptome sequencing suggested that the expression of CST1 was significantly increased in metastatic cancer, and high CST1 expression was correlated with a worse prognosis. Our data further confirmed that the overexpression of CST1 may significantly promote the migration and invasion of gastric cancer cells in vitro and enhance liver, lung, and peritoneal metastasis of gastric cancer in nude mice. Meanwhile, high expression of CST1 promoted the epithelial-mesenchymal transition (EMT) of gastric cancer cells. Mechanistically, a co-immunoprecipitation experiment combined with mass spectrometry analysis confirmed that CST1 could interact with GPX4, a key protein regulating ferroptosis. CST1 relieves GPX4 ubiquitination modification by recruiting OTUB1, improving GPX4 protein stability and reducing intracellular reactive oxygen species (ROS), thereby inhibiting ferroptosis and, in turn, promoting gastric cancer metastasis. Moreover, clinical data suggested that CST1 is significantly increased in peripheral blood and ascites of gastric cancer patients with metastasis; multivariate Cox regression model analysis showed that CST1 was an independent risk factor for the prognosis of gastric cancer patients. Overall, our results elucidated a critical pathway through which high CST1 expression protects gastric cancer cells from undergoing ferroptosis, thus promoting its progression and metastasis. CST1 may be used as a new oncological marker and potential therapeutic target for gastric cancer metastasis.

## Introduction

Gastric cancer (GC) is a malignant tumor of the digestive tract. It is the fifth most common neoplasm and the fourth most deadly tumor worldwide [1]. Metastasis is an important factor affecting the prognosis of gastric cancer patients. Yet, the underlying mechanism of gastric cancer metastasis is still unclear. Chemotherapy has been the most common treatment approach for metastatic gastric cancer patients for years; however, recent data have suggested that the overall curative effect and prognosis after chemotherapy are still poor [2]. Consequently, searching for novel treatment methods for patients with gastric cancer metastasis is of utmost importance.

Over the last decade, molecular targeted therapy has emerged as a new treatment for malignant tumors. Cystatin (CST) is a class of proteins widely distributed in human body fluids and tissues that inhibit cysteine proteases and can be divided into three types [3]. Cystatin SN is a secreted peptide encoded by the *CST1* gene with a relative molecular mass of about 14KD. It belongs to the type 2 family of the cysteine protease inhibitor superfamily [4]. Cystatins domain consisting of 100 amino acid residues can bind to the active site of cysteine protease, thereby inhibiting the hydrolysis activity of cysteine protease [5, 6]. CST1 protein is mainly distributed in the submandibular gland, gallbladder, and uterus, but it also highly expresses malignant tissue [7]. Recent studies have suggested that the overexpression of CST1 participates in the proliferation, invasion, and metastasis of lung cancer, breast cancer, colorectal cancer, and other tumors [8–10]. Kim et al. found that CST1 upregulation might be involved in colorectal tumorigenesis by neutralizing the inhibition of CTSB proteolytic activity by CST3 [11]. Interestingly, high expression of CST1 reduces auranofin-induced cell death by inhibiting intracellular reactive oxygen species (ROS) generation in CRC cells [12]. Moreover, some data suggested that CST1 is involved in cathepsin inhibition in gastric cancer, promoting its progression by regulating transcription factor HOXC10 and the Wnt signaling pathway [13–15]. However, the molecular mechanism of CST1 promoting the malignant progression of gastric cancer needs to be further explored.

Ferroptosis is a new type of cell death that results from iron-dependent lipid peroxide accumulation. During ferroptosis, reactive oxygen species (ROS) production is increased, mitochondrial volume is reduced, and membrane density is increased [16, 17]. A critical role of ferroptosis in tumor metastasis has been gradually revealed. For example, lymphoid tissue protects tumor cells against ferroptosis and promotes melanoma metastasis [18]. Moreover, in a spontaneous mouse model of HER2-positive breast cancer, induction of ferroptosis suppresses tumor brain metastasis [19].

Recent data has revealed that GPX4, a phospholipid hydroperoxide glutathione peroxidase, contributes to ferroptosis, thus promoting cancer behavior. The function of GPX4 is to maintain intracellular redox homeostasis by inhibiting lipid peroxidation and protecting cells from death caused by membrane lipid peroxidation [20]. Previous studies have shown that GPX4 is highly expressed in metastatic cancers and is closely related to tumor progression [21, 22]. Lu et al. discovered that KLF2 inhibits cell renal cell carcinoma migration and invasion by regulating ferroptosis through GPX4 [23]. However, the role of GPX4-ferroptosis in gastric cancer metastasis remains unclear.

The ubiquitin-proteasome system (UPS) mediates 80 to 85% of protein degradation in eukaryotes. The UPS system involves ubiquitin molecules, substrate proteins, ubiquitin-activating enzyme (E1), ubiquitin-conjugating enzyme (E2), ubiquitin ligase (E3), deubiquitinase (DUB), and the proteasome. Some studies suggested that the dysregulation of this system is closely related to tumor occurrence and metastasis [24, 25]. Studies have also shown that UPS regulates different aspects of ferroptosis [26, 27]. For example, DMOCPTL, a derivative of the natural product parthenolide, targets GPX4, increases GPX4 ubiquitination modification, and promotes GPX4 degradation [28]. So far, a few deubiquitinases or ubiquitin ligases targeting GPX4 have been reported.

In this study, we explored the role of cysteine protease inhibitor SN in promoting gastric cancer metastasis. We hypothesized that CST1 could regulate gastric progression by regulating GPX4 and ferroptosis.

## Results

### CST1 is up-regulated in primary and metastatic GC and is associated with a poor prognosis

**Fig. 1:**
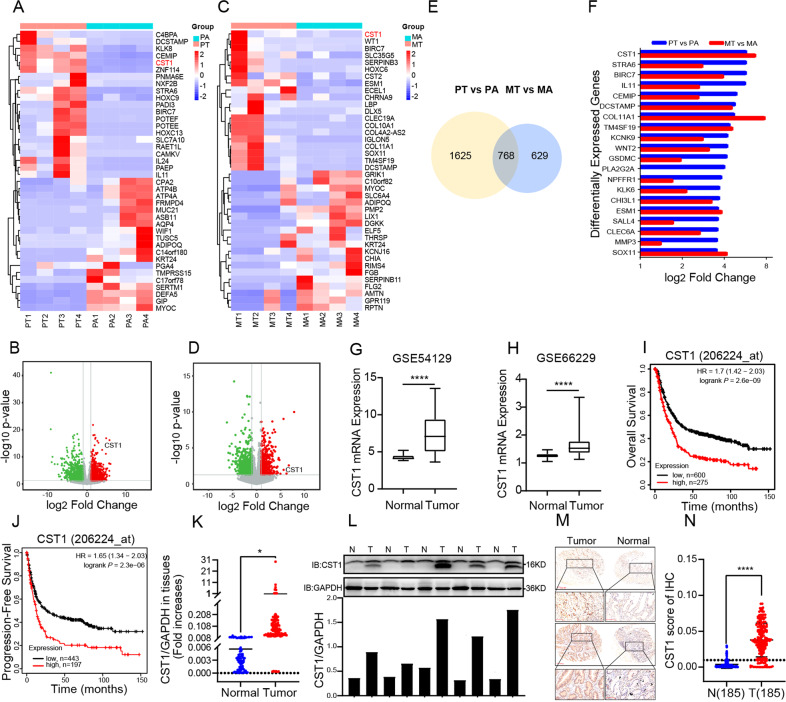
CST1 is up-regulated in primary and metastatic GC tissues and is associated with a poor prognosis. Heatmap mainly showing expression levels of 20 up-regulated and 20 down-regulated different expressed genes in primary GC (**A**) and metastatic GC (**C**) vs. adjacent normal tissues (>1.5-fold). Volcano plots with differentially expressed genes in primary GC (**B**) and metastatic GC (**D**) vs. adjacent normal tissues. **E** Venn diagram representation of 768 overlapped up-regulated genes. **F** Histogram was shown the overlapped highest 20 up-regulated genes in these two clusters. **G**, **H** Higher expression of CST1 was found in GC samples than the matched normal tissues (based on GSE54129 and GSE66229 database). **I**, **J** Kaplan–Meier plots of overall survival and progression-free survival for GC samples from the KM Ploter database. **K** RT-qPCR analysis of CST1 mRNA expression in 100 pairs of GC patient samples. Data are shown as the mean ± SD of triplicate independent sets of experiments; statistical significance was assessed by paired *t*-test, *<0.05, *n* = 100. **L** Western blot analysis was performed using an antibody against CST1 in 5 pairs of GC patients’ samples (upper panel); protein band intensities were measured by ImageJ software and normalized to GAPDH (lower panel). **M** IHC staining was performed using an antibody against CST1 and representative photographs of CST1 in GC patients. Scale bar: 100 μm. **N** IHC stain scoring of CST1 in 185 GC tissues and 185 normal tissues, statistical significance was assessed by unpaired *t*-test, ****<0.0001.

To identify the differential genes associated with the peritoneal metastasis of GC, we first performed RNA-seq analysis to compare their expression levels between 4 paired primary GC tissues and adjacent normal tissues. Genes with a *p* value <0.05 were only considered. The RNA-seq results revealed 3904 different genes, and the heatmap showed the highest and lowest 20 expression genes (Fig. 1A). Among differentially expressed genes, CST1 was significantly up-regulated in primary GC tissues. Volcano plots showed consistent results (Fig. 1B).

Then, we performed RNA-seq analysis again in 4 paired peritoneal metastasized GC tissues. The corresponding controls, heatmap, and volcano plots showed that CST1 was also up-regulated in this cohort (Fig. 1C, D). Then, the Venn diagram of the up-regulated genes showed the numbers of common and specific genes in two clusters, screening 768 common up-regulated genes. CST1 was up-regulated in GC and peritoneal metastasized GC tissues (Fig. 1E, F).

To validate our results, we evaluated the expression levels of CST1 in the matched pairs of GC and normal tissues samples using the GEO RNA-seq database, including GEO GSE54129, and GSE66229 database (Fig. 1G, H), GEO GSE79973, GSE26899, GSE13911, GSE19826 database (Fig. S1A), and TCGA database (Fig. S1B), consistent with our observation, the result from these datasets showed significantly up-regulated CST1 expression in GC tissue samples. Additionally, the expression of CST1 was positively correlated with the clinical stage of gastric cancer, but the difference was not statistically significant (Fig. S1C). More interestingly, overall survival and progression-free survival were lower in patients with higher CST1 levels in the Kaplan-Meier Plotter online database (Fig. 1I, J).

To further test the expression of CST1 in GC tissues, RT-qPCR was performed on 100 paired GC and adjacent normal tissues, showing that CST1 was up-regulated in GC tissues (Fig. 1K). In addition, Western blot analysis of 5 paired GC and adjacent normal tissues revealed that CST1 expression was higher in GC than in paired normal tissues (Fig. 1L). At the same time, the immunohistochemical analysis result showed that the CST1 was highly expressed in the GC tissues regardless of their differentiation grade (Fig. 1M); the upper panel was poorly differentiated while the down panel was well differentiated. In addition, IHC of 185 paired GC and normal tissues revealed that CST1 was highly expressed in GC tissues (Fig. 1N).

### CST1 promotes GC cell metastasis but has no effect on cell proliferation in vitro

**Fig. 2:**
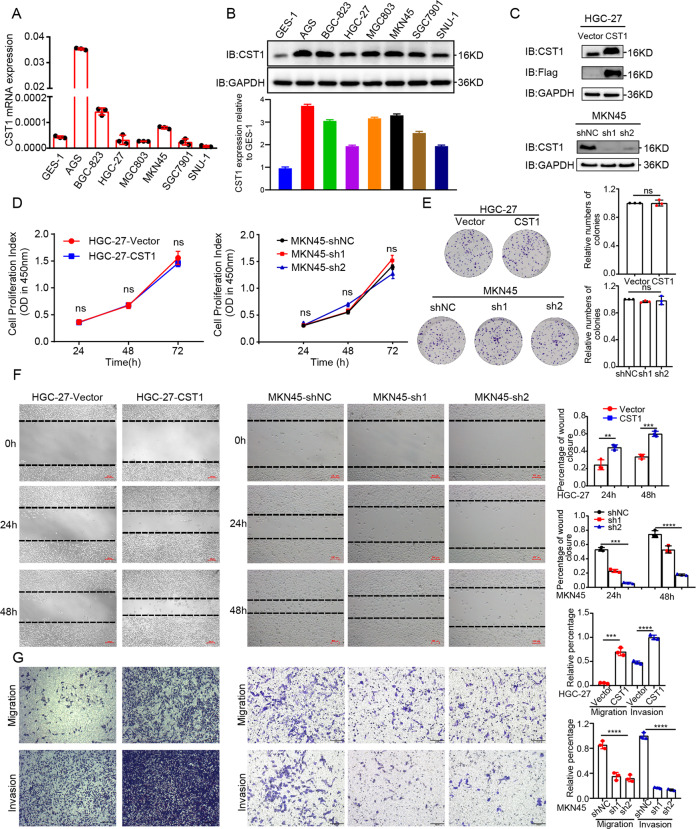
CST1 promotes GC cell metastasis but not proliferation in vitro. **A**, **B** RT-qPCR and Western blot analysis showing the expression of CST1 in different GC cell lines. Total GAPDH was used as a loading control. **C** Western blot analysis of HGC-27 and MKN45 stably transfected with CST1 overexpression/knockdown lentiviruses and control lentiviruses. Total GAPDH was used as a loading control. **D** CCK8 assay analyzed the proliferation of HGC-27-Vector/HGC-27-CST1 and MKN45-shNC/MKN45-sh1-CST1/MKN45-sh2-CST1 stable cell lines. Data are shown as the mean ± SD of triplicate independent sets of experiments; statistical significance was assessed by paired *t*-test. **E** A colony formation assay. Left panel: representative images, right panel: quantification analysis. Data from independent experiments are presented as the mean ± SD. Statistical was assessed by unpaired *t*-test, ns means no significance. **F** Wound healing analysis for assessing migration of HGC-27-Vector/HGC-27-CST1 and MKN45-shNC/MKN45-sh1-CST1/MKN45-sh2-CST1 at 0, 24, and 48 h. Representative images (left panel) and quantification (right panel) are shown as indicated. Data from independent experiments are presented as the mean ± SD. Statistical significance was assessed by an unpaired *t*-test. ***p < 0.001. Scale bar: 100 μm. **G** Transwell migration and Matrigel invasion assays were performed to assess migration and invasion ability of CST1-overexpression and knockdown stable cell lines. Representative images (left panel) and quantification (right panel) are shown as indicated. Data from independent experiments are presented as the mean ± SD. Statistical significance was assessed by an unpaired *t*-test. ***p < 0.001. Scale bar: 100 μm.

Next, we examined the CST1 mRNA and protein expression levels in GC cell lines. Higher expression of CST1 mRNA was found in AGS, BGC823, and MKN45 cell lines than in HGC-27 and SNU-1 cell lines (Fig. 2A, B). We then used HGC-27 cells to stably overexpress CST1, AGS and MKN45 cells to knockdown CST1 (Fig. 2C and S2A) and to examine the role of CST1 on cell behavior in vitro. CCK8 and plate clone formation assays suggested that the overexpression of CST1 had no effect on HGC-27 cells compared to control cells; similar results were obtained for MKN45-sh1-CST1 and MKN45-sh2-CST1 vs. control cells and CST1 knockdown AGS stable cell lines (Fig. 2D, E and S2B, C). However, the wound healing assay indicated that CST1 could promote cell migration. HGC-27-CST1 cells showed faster migration while MKN45-sh1-CST1/MKN45-sh2-CST1 cells and CST1 knockdown AGS stable cell lines showed slower migration compared to the negative control group (Fig. 2F and S2D).

To further assess the contribution of CST1 to the development of migratory and invasive phenotypes of GC cells, migration and invasion experiments were conducted using both HGC-27 and MKN45 and AGS cells. Data indicated that the overexpression of CST1 significantly increased the migration and invasion of HGC-27 cells compared with its control, while the migration of MKN45 and AGS cells was reduced (Fig. 2G and S2E). Collectively, these observations suggest that CST1 is a positive regulator of migration and invasion in GC cells.

### CST1 interacts with GPX4 to improve the stability of the GPX4 protein

**Fig. 3:**
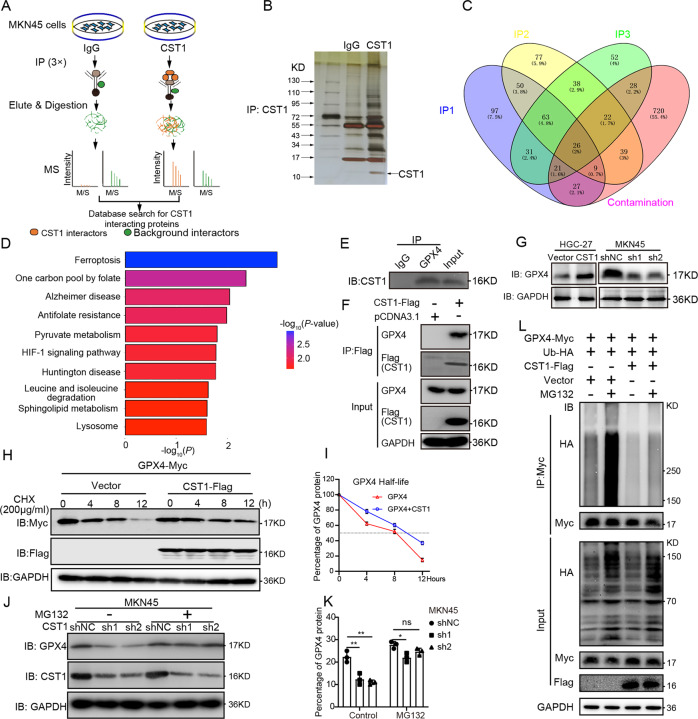
CST1 interacts with GPX4 to improve the stability of GPX4 protein. **A** Schematic of CST1 interactor discovery. The potential interactors were presented at higher levels in the CST1 experimental group than in the IgG control group. Three replicates of IP-MS were conducted. **B** Silver staining of IP cell lysates. **C** Venn diagram of three times of LC-MS results showing 63 co-upregulated proteins. **D** KEGG pathways analysis of 63 co-upregulated proteins. **E** Immunoprecipitation of the CST1 protein by an anti-GPX4 antibody in MKN45 cells. IgG was used as a negative control. **F** Immunoprecipitation of the GPX4 protein by an anti-Flag antibody in HEK293 cells transfected with pcDNA3.1-FLAG-CST1. PcDNA3.1-vector was used as a negative control. **G** Western blot showing GPX4 expression in HGC-27 and MKN45 stable cell lines; total GAPDH was used as a loading control. **H**, **I** Degradation of the GPX4 protein was measured after the treatment of 200 µg/ml CHX at the indicated time points in HEK293T, which transfected with Flag-tagged CST1 expression plasmids and Myc-tagged GPX4 expression plasmids. **J**, **K** Western blot analysis of GPX4 expression after treatment with 20 µM MG132 for 4 h in MKN45 stable cell lines. Data were expressed as a fold-change relative to control. **L** Analysis of GPX4 ubiquitination was performed by immunoprecipitation using an anti-Myc antibody, followed by immunoblot with anti-HA antibody and anti-Myc antibody in HEK293T cells transfected with the indicated constructs.

To investigate the mechanism underlying the CST1 in GC cells, we analyzed MKN45 cells by immunoprecipitation (IP) assay using CST1-specific antibody (IgG antibody was used as a control group). The samples were then separated using SDS-PAGE and analyzed by a liquid chromatograph-mass spectrometer (LC-MS); the experiment was performed three times (Fig. 3A). Silver staining of the IP cell lysates revealed that CST1 was successfully pulled down and further to LC-MS (Fig. 3B). Venn diagram revealed that 63 proteins were enriched by CST1 antibody compared to those in the IgG control samples (Supplementary File 4). The data of the three time-mass spectrometry eliminated the contamination proteins (Fig. 3C). Kyoto Encyclopedia of Genes and Genomes (KEGG) pathways analysis of these 63 proteins further revealed that the ferroptosis pathway was highly enriched (Fig. 3D) and GPX4 was a key protein in the ferroptosis pathway.

To further demonstrate the protein-protein interaction between CST1 and GPX4, we first performed Co-IP experiments using the MKN45 cells overexpressing CST1 (Fig. 3E). Consequently, a strong binding between CST1 and GPX4 was found. Next, the HEK293T cells were transiently transfected with Flag-tagged CST1 expression plasmids, followed by Co-IP assays with an anti-Flag antibody. The result showed that CST1 and GPX4 proteins bound to each other in these cells (Fig. 3F). Collectively, these observations suggested that CST1 interacting with GPX4 may induce the malignant progression of GC.

Next, we examined whether CST1 modulates the mRNA or protein levels of GPX4 through their interaction with each other. To test this hypothesis, HGC-27-Vector/HGC-27-CST1 and MKN45-shNC/MKN45-sh1-CST1/MKN45-sh2-CST1 cells were established. The levels of CST1 and GPX4 expression were confirmed by RT-qPCR analysis and Western blot. The results showed that CST1 overexpression increased GPX4 protein levels (Fig. 3G) without altering GPX4 mRNA level (Fig. S3A). Moreover, in the GEO database GSE54129 and GSE66229 data sets, the Pearson correlation line analysis showed no significant correlation between CST1 and GPX4 gene expression (the *p* values were all >0.05) (Fig. S3B, C).

Next, we examined whether CST1 enhances the stability of GPX4 protein. Cycloheximide (CHX), a protein synthesis inhibitor, has been used to determine the effect of CST1 on GPX4 stability. The HEK293T cells were transiently transfected with Flag-tagged CST1 expression plasmid and Myc-tagged GPX4 expression plasmid and treated with 200 µg/ml CHX for different times to block protein synthesis. The degradation rates of the existing GPX4 protein were measured by Western blot. The results showed that the overexpression of CST1 weakened GPX4 degradation compared with the control group (Fig. 3H, I). To further evaluate the relationship between GPX4 protein stability mediated by CST1 and the proteasome system, we used MG132, a 26S proteasome inhibitor. Notably, treatment with MG132 allowed an accumulation of GPX4 protein, as shown in MKN45-shNC/MKN45-sh1-CST1/MKN45-sh2-CST1 cells. Also, GPX4 protein levels induced by proteasome inhibition could not be further decreased by CST1 knockdown (Fig. 3J, K).

Taken together, our data indicate that CST1 upregulates GPX4 expression by increasing the stability of the GPX4 protein through the proteasome pathway.

### CST1 relieves GPX4 ubiquitination through deubiquitinase OTUB1

To study whether CST1 affects GPX4 ubiquitination, we conducted ubiquitination assays. We performed ubiquitination assays in HEK293T that induced exogenous Myc-GPX4 and HA-Ub in the presence or the absence of Flag-CST1 and/or MG132. The cell extracts were immunoprecipitated using an anti-Myc antibody, followed by immunoblot analysis with an anti-HA antibody. As expected, GPX4-ubiquitination was high without CST1 (Fig. 3L, lane 2); however, the existence of CST1 obviously decreased the ubiquitination of GPX4 (Fig. 3L, lane 3–4); and GPX4-ubiquitination was lower in the presence of CST1 without MG132 (Fig. 3L, lane 3). To explore the effect of CST1 on the ubiquitination of endogenous GPX4 in gastric cancer cells, we used the CST1 knockdown stable cell line MKN45-shNC/MKN45-sh1-CST1/MKN45-sh2-CST1, immunoprecipitated GPX4, respectively, and then detected the Ub level by WB. The results showed that in the negative control group, the level of ubiquitination of GPX4 was low, whereas the level of ubiquitination of GPX4 was increased in CST1-knockdown MKN45-sh1-CST1/MKN45-sh2-CST1 cells (Fig. S3D). These findings suggest that CST1 inhibits the ubiquitination of GPX4 to regulate GC progression.

**Fig. 4:**
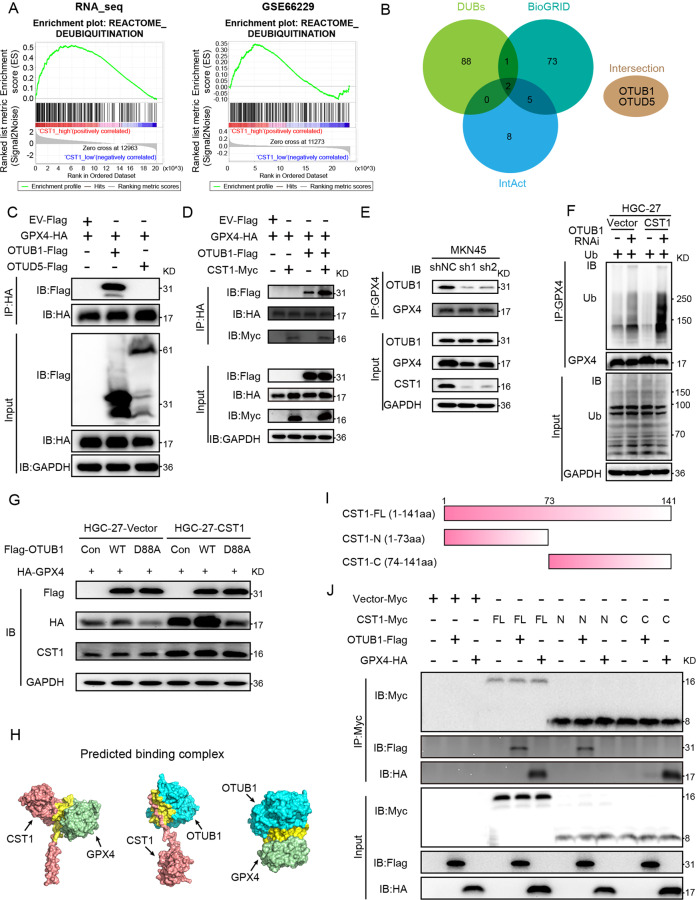
CST1 relieves GPX4 ubiquitination through deubiquitinase OTUB1. **A** GSEA analysis of our previous transcriptome sequencing data and GEO data set (GSE66229) enriched the deubiquitination pathway. **B** Predicting the deubiquitinase DUBs that interact with GPX4 through the online database (BioGRID, IntAct) and Venn analysis with all known DUBs. Intersection proteins include OTUB1 and OTUD5. **C** Co-IP assay on HEK293T cells transfected with Flag-tagged OTUB1 and OTUD5 plasmids. **D** WB detection of Flag, HA, Myc tagged proteins after IP in cells co-transfected with Myc-tagged CST1 and Flag-tagged OTUB1. **E** Endogenous Co-IP of GPX4 in MKN45-shNC/MKN45-sh1-CST1/MKN45-sh2-CST1. WB detection of IP proteins; OTUB1 binding to GPX4 was significantly reduced. **F** HGC-27-Vector/HGC-27-CST1 cells were transiently transfected with OTUB1 siRNA. The ubiquitination assay showed that the level of ubiquitin bound by GPX4 increased with the decrease of OTUB1, and the ubiquitination level of GPX4 was more obvious in HGC27 cells overexpressing CST1. **G** HGC-27-Vector/HGC-27-CST1 cells were transiently transfected with Flag-OTUB1-Con/WT/D88A and HA-GPX4 plasmids, WB detection showed that the GPX4 protein in the OTUB1-WT group was more stable, while the GPX4 protein in the OTUB1-D88A group was reduced. **H** Predicted binding complex models of CST1, OTUB1 and GPX4 by using the Cluspro online protein docking tool. **I** Schematic diagram of the construction of full-length and truncated CST1 proteins. **J** HEK293T cells were transfected with CST1-FL/N/C-Myc plasmids and OTUB1-Flag, GPX4-HA plasmid, respectively. Myc protein was immunoprecipitated, WB showed that CST1-FL and CST1-N truncated protein can bind to OTUB1, while CST1-FL and CST1-C truncated proteins can bind to GPX4. The SDS-PAGE used for separating CST fragments was 15%. (GSEA Gene set enrichment analysis, Con control/empty plasmid, WT Wild-type plasmid, D88A the D88 site of OTUB1 is point mutated to A plasmid).

Next, we investigated the deubiquitinase or E3 interacting with GPX4. First, we performed Gene Set Enrichment Analysis (GSEA) on the pre-GC tissue transcriptome sequencing data and GEO dataset (GSE66229), according to the CST1 gene expression, the two groups of data were divided into CST1 high and low expression groups respectively. The results showed that in our transcriptome sequencing data, the genes in the CST1 high expression group were more involved in the deubiquitination modification pathway (Fig. 4A left); Similarly, in the GSE66229 dataset, genes in the CST1 high expression group are also mainly involved in the deubiquitination pathway (Fig. 4A right); the above bioinformatics analysis results confirm that CST1 is involved in protein deubiquitination modification process.

By predicting the deubiquitinase DUBs that interact with GPX4 through the online database (BioGRID, IntAct) and performing Venn analysis with all known DUBs, we found that OTUB1 and OTUD5 proteins may potentially interact with GPX4 (Fig. 4B). To verify which deubiquitinase binds to GPX4, we transfected Flag-tagged OTUB1 and OTUD5 plasmids into HEK293T cells, respectively, and performed Co-IP experiments with HA-GPX4. The results indicated that only OTUB1 could bind to GPX4 (Fig. 4C). Next, we co-transfected Myc-tagged CST1 and Flag-tagged OTUB1, the results showed that in the absence of OTUB1, CST1 could bind to GPX4 (Fig. 4D, lane 2); in the absence of CST1, OTUB1 could also bind to GPX4 (Fig. 4D, lane 3); and when OTUB1-Flag was transfected at the same time, OTUB1 bound to GPX4 was significantly increased upon CST1-Myc (Fig. 4D, lane 4).These results implied that CST1 mediates the deubiquitination of GPX4 by OTUB1. To further verify this, we first performed endogenous Co-IP in a cell line in which CST1 was stably knocked down by MKN45. We found that with the decline of CST1, OTUB1 decreased the binding to GPX4 (Fig. 4E). HGC-27 cells were transiently transfected with OTUB1 siRNA, and the results showed that the level of ubiquitin bound by GPX4 increased with the decrease of OTUB1 and the ubiquitination level of GPX4 was more obvious in HGC-27 cells even overexpressing CST1 (Fig. 4F).

OTUB1 is an important deubiquitinase, which mainly depends on the inhibition of E2-conjugating enzymes and can stabilize the target proteins. When the D88 site of OTUB1 is point mutated to A(D88A), the deubiquitinase activity of OTUB1 can be significantly attenuated [27, 29]. To address whether CST1-stabilizing GPX4 protein dependent on OTUB1 deubiquitinase activity. We constructed the D88A mutant of OTUB1, and then transfected to HGC-27-Vector/CST1 stable cells, the results showed that the GPX4 protein in the OTUB1-WT group was more stable, while the GPX4 protein in the OTUB1-D88A group was significantly reduced, and this change was more pronounced after CST1 overexpression (Fig. 4G). The results confirmed that CST1 mediates the effect of OTUB1 to stabilize GPX4 protein, and depends on the inhibition of E2-conjugating enzymes by OTUB1 to exert its deubiquitin function.

Subsequently, we used the method of protein-protein docking to predict the 3D complex model and potential interaction domains of CST1, OTUB1 and GPX4. The 3D spatial structures of these proteins were obtained from SWISS-MODE and PDB databases (Fig. S4A), and the most likely complex model of CST1, OTUB1 and GPX4 binding was predicted using the Cluspro online protein docking tool (Fig. 4H). Then PDBePISA was used online, the interaction surface in the protein complex was analyzed, and it was found that the free energy of binding between CST1, OTUB1 and GPX4 was low (Fig. S4B), indicating that a stable complex can be formed between them. LIGPLOT was used to map the “eyelash figure” of protein-protein interactions (Fig. S4C), showing the potential binding domains between CST1, OTUB1 and GPX4.

Further, we constructed different truncated proteins of CST1. The full length of CST1(CST1-FL) protein contains 141 amino acids, of which amino acids 1-73 truncation name as CST1-N, and amino acids 74-141 truncation name as CST1-C (Fig. 4I), which were added Myc tags respectively. Subsequently, HEK293T cells were transfected with CST1-FL/N/C-Myc plasmids and OTUB1-Flag, GPX4-HA plasmid, respectively. Then Myc-protein was immunoprecipitated, we found that CST1-FL and CST1-N truncated protein can bind to OTUB1, while CST1-FL and CST1-C truncated proteins can bind to GPX4 (Fig. 4J). The above results indicate that OTUB1 and GPX4 can combine with different domains of CST1 to form a stable complex structure.

### CST1 reduces intracellular ROS and inhibits ferroptosis through GPX4

**Fig. 5:**
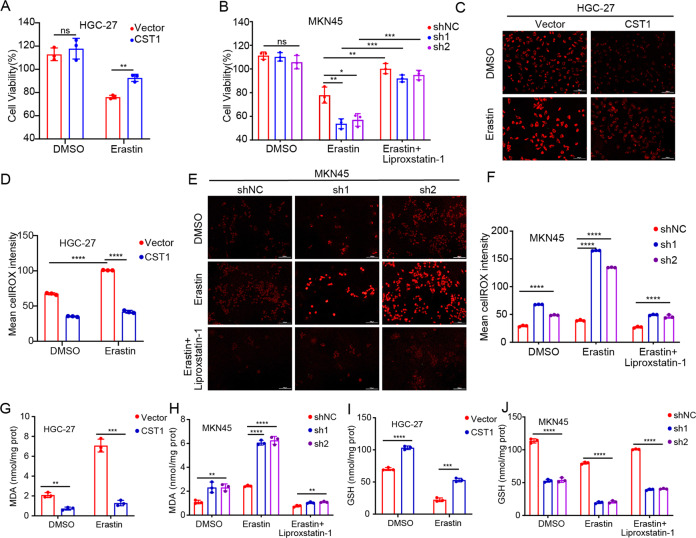
CST1 reduces intracellular ROS and inhibits ferroptosis through GPX4. **A** Treatment of HGC-27-Vector/HGC-27-CST1 with the ferroptosis inducer erastin (10 μM) inhibited the decrease of HGC-27-CST1 cell viability relative to the DMSO-treated group (****<0.0001). **B** Treating MKN45-shNC/MKN45-sh1-CST1/MKN45-sh2-CST1 cells with erastin (10 μM) decreased the viability more significantly than the DMSO-treated group (****<0.0001). After ferroptosis inhibitor liproxstatin-1 treatment, the viability of MKN45-sh cells increased. **C**, **D** The level of ROS in HGC-27-Vector/HGC-27-CST1cells detected by fluorescence microscopy. The results showed that in the erastin-treated group, CST1 reduced the level of intracellular ROS (****<0.0001). **E**, **F** In MKN45-shNC/MKN45-sh1-CST1/MKN45-sh2-CST1 cells, intracellular ROS was significantly increased after erastin treatment (****<0.0001), while ferroptosis inhibitor liproxstatin-1 reversed this process. **G** MDA content in HGC-27-CST1 cells decreased, and this difference was more significant after erastin treatment (****<0.0001). **H** MDA content in MKN45-sh cells significantly increased, and the difference was more significant after erastin treatment. After liproxstain-1 treatment, the MDA content decreased again. **I** The GSH content in HGC-27-CST1 cells increased. **J** GSH content in MKN45-sh cells decreased, and the difference was more significant after erastin treatment and increased after liproxstatin-1 treatment (****<0.0001).

Knowing that CST1 can regulate the stability of GPX4 protein, and GPX4 is a key molecule in regulating ferroptosis, we investigated whether CST1 affects the occurrence of ferroptosis through GPX4. Treatment of HGC-27-Vector/ HGC-27-CST1 cells with the ferroptosis inducer erastin (10 μM) inhibited the decrease of HGC-27-CST1 cell viability relative to the DMSO-treated group (Fig. 5A). Moreover, treating MKN45-shNC/MKN45-sh1-CST1/MKN45-sh2-CST1 cells with erastin (10 μM) decreased the viability of cells more significantly than the DMSO-treated group. At the same time, the ferroptosis inhibitor liproxstatin-1 (1 μM) was used for the recovery experiment, and the results showed that the viability of MKN45-sh cells was increased (Fig. 5B). The results suggested that CST1 might be associated with erastin-induced ferroptosis.

The level of ROS in HGC-27-Vector/HGC-27-CST1 cells was further detected by fluorescence microscopy. The results showed that in the erastin-treated group, CST1 reduced the level of intracellular ROS (Fig. 5C, D). In MKN45-shNC/MKN45-sh1-CST1/MKN45-sh2-CST1 cells, intracellular ROS was significantly increased after erastin treatment while ferroptosis inhibitor liproxstatin-1 rescued the above effect (Fig. 5E, F).

Next, we detected the content of glutathione (GSH), an important substrate of GPX4, and malondialdehyde (MDA), a lipid peroxide product. The results showed that the MDA content in HGC-27 cells decreased with the up-regulation of CST1 expression, and this difference was more significant after erastin treatment (Fig. 5G). However, after CST1 was down-regulated, the MDA content in MKN45 cells significantly increased, and the difference was more significant after erastin treatment. After liproxstain-1 treatment, the MDA content decreased again (Fig. 5H). The GSH content in HGC-27 cells increased with the up-regulation of CST1 expression, while the GSH content in MKN45 cells significantly decreased after CST1 down-regulation, and the difference was more significant after erastin treatment and increased after liproxstatin-1 treatment (Fig. 5I, J).

Intracellular iron overload is also one of the important signs of ferroptosis. Thus, we detected the intracellular iron level by Prussian blue staining. The results showed that the up-regulation or down-regulation of CST1 did not significantly affect the intracellular iron level (Fig. S5A, B).

The above experimental results confirmed that CST1 could reduce intracellular ROS, and thus inhibit the occurrence of ferroptosis.

### CST1 mediate GPX4 protein stability to promote migration and invasion in epithelial-mesenchymal transition manner in GC cells

**Fig. 6:**
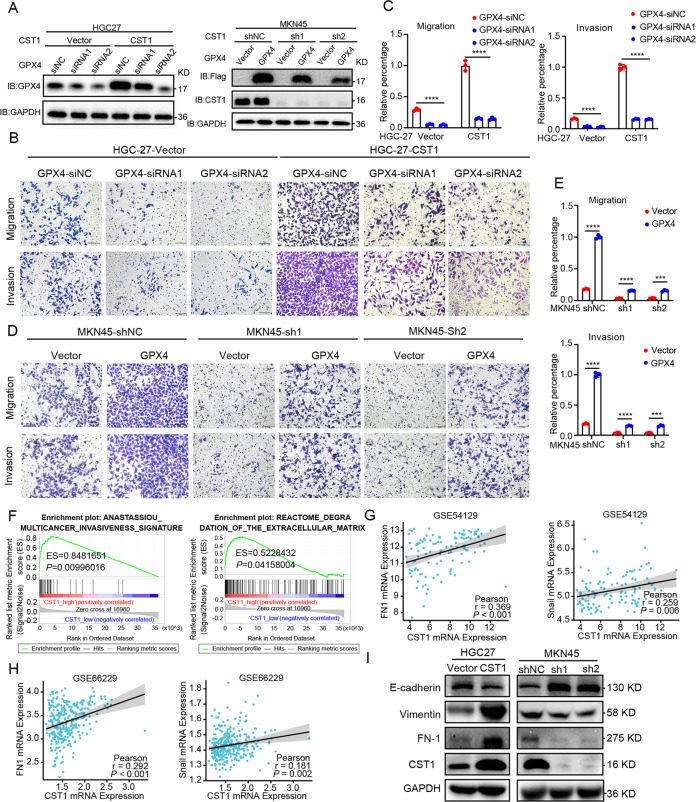
CST1 mediates GPX4 protein stability to promote migration and invasion in epithelial-mesenchymal transition manner in GC cells. **A** Western blot analysis of GPX4 in HCG27 and MKN45 stable cell lines transfected with GPX4-siRNA or exogenous overexpressing GPX4. **B**, **C** Transwell migration and invasion assays were performed in HGC-27-Vector/HGC-27-CST1 treated with GPX4-siRNA and control as indicated. **D**, **E** Transwell migration and invasion assays were performed in MKN45-shNC/MKN45-sh1-CST1/MKN45-sh2-CST1 treated with transient transfection of GPX4 exogenous overexpression or vector control as indicated. Data are presented as the mean ± SD. Statistical significance was assessed by an unpaired *t*-test. ****p < 0.0001. **F** Gene set enrichment analysis of CST1 related to invasiveness and degradation of the extracellular matrix. **G**, **H** Correlation analysis of CST1 and FN1/Snail in GSE54129 and GSE66229 as illustrated in the dot plot (Person’s correlation test). **I** Western blot analysis of E-cadherin, Vimetin, FN1 expression in the indicated cells.

To assess whether GPX4 is an effective target of CST1, knockdown of GPX4 in HGC-27-Vector and HGC-27-CST1 cells were used and analyzed by Western blot, which allowed for the determination of the transient transfection efficiency of GPX4-siRNA1 and GPX4-siRNA2. On the contrary, exogenous overexpression of GPX4 was found in MKN45-shNC, MKN45-sh1-CST1, and MKN45-sh2-CST1 cells, and Western blot revealed the transfection efficiency (Fig. 6A). Transwell assays were performed using HGC-27 and MKN45 cells to investigate the effect of GPX4 on cell migration and invasion abilities. The results showed that compared to HGC-27-CST1, GPX4-siRNA1 and GPX4-siRNA2 cohort significantly decreased the migration ability of HGC-27-CST1 cells. However, overexpression of GPX4 in MKN45-sh1-CST1 and sh2-CST1 cells increased the migration and invasion ability (Fig. 6B, D). The migration/invasion rate in relevant HGC-27 and MKN45 groups further confirmed this data (Fig. 6C, E).

Epithelial-mesenchymal transitions (EMT) have an important role in conferring the ability of tumor cells to migrate, invade, and metastasize [30]. CST1 can promote EMT in thyroid cancer and liver cancer [31, 32]; however, its role in promoting EMT in gastric cancer has not yet been investigated. To explore the signaling pathways by which CST1-GPX4 exerted its migration and invasion effects, we performed RT-qPCR assays to measure diverse pathways. CST1 overexpression promoted HGC-27 cell mesenchymal marker (N-Cadherin) and Snail mRNA expression and reduced the epithelial marker (E-Cadherin) mRNA expression. After down-regulation of CST1 expression, the mRNA expression of mesenchymal markers (N-Cadherin) and Snail of MKN45 decreased, while the mRNA expression of epithelial markers (E-Cadherin) increased (Fig. S6A). We also found that CST1 was positively associated with the EMT marker.

To verify this mechanism, we performed Gene set enrichment analysis showed that the gene sets related to invasiveness and degradation of the extracellular matrix were enriched in samples with high CST1 expression (Figs. 6F and S6B). To further define the role of CST1 and EMT in GC cells, we evaluated their expression in GEO GSE54129, and GSE66229. Statistical analysis revealed a significant positive correlation when the expression level of CST1 in these two GEO datasets was plotted against that of FN1 (*p* < 0.001), Snail (*p* < 0.01), MMP9 (*p* < 0.001); however, there was no significant correlation between CST1 and E-cadherin, Vimentin (Figs. 6G, H and S6C). This observation was further confirmed by alterations in the protein expression patterns of epithelial and mesenchymal markers, which was consistent with the mRNA expression in the GEO dataset. Briefly, CST1 overexpression promoted EMT, as evidenced by suppression of E-cadherin (epithelial marker) and the upregulation of Vimentin/FN1 (mesenchymal marker) in HGC-27 cells. Conversely, MKN45-sh1/2-CST1 cells reverted to an epithelial phenotype compared with control cells (Fig. 6I). Together, these observations demonstrated that CST1 is a positive regulator of EMT in GC cells.

### CST1 promotes gastric cancer cell migration and invasion by regulating GPX4-K11 site ubiquitination

It is a key experiment to explore the regulation of GPX4 stabilization by CST1 by mutating the ubiquitination site of GPX4 and promoting gastric cancer migration and invasion. To search for GPX4 ubiquitination sites, we firstly adopted bioinformatics methods, respectively, in GPS-Uber (http://gpsuber.biocuckoo.cn/index.php) and BDM-PUB (http://bdmpub.biocuckoo.org/prediction.php) online website predicted the possible ubiquitination site of GPX4, and found that the K11 site could be predicted in both sites (Fig. S7A), so we assumed the K11 amino acid of GPX4 as a potential ubiquitination site of transformation. Next, we constructed a GPX4-K11 site mutant, transfected HGC-27-Vector/HGC-27-CST1 stable gastric cancer cells with GPX4-WT plasmid, empty control plasmid and Ub-HA plasmid, respectively, and immunoprecipitated Myc tag. The ubiquitination level of the precipitated protein was detected by WB. The results showed that in HGC-27-Vector cells with low CST1 protein, the ubiquitination level of GPX4 protein was significantly decreased after the GPX4-K11 site mutation (Fig. S7B lane 3). In HGC-27-CST1 cells with up-regulated CST1, the ubiquitination level of GPX4 protein decreased more significantly after GPX4-K11 site mutation (Fig. S7B lane 6). The above results confirm that the GPX4-K11 site is its ubiquitination site, but it is worth noting that after mutating the K11 site, GPX4 still undergoes a small amount of ubiquitination modification, indicating that there are other possible ubiquitination sites, further research is needed.

Further, we investigated whether GPX4-K11 ubiquitination site mutation affects the migration and invasion ability of gastric cancer cells, and found that in HGC-27-Vector cells with low CST1 protein, after GPX4-K11 site mutation, the migration and invasion abilities of HGC-27 cells were enhanced; in HGC-27-CST1 cells with up-regulated CST1, after GPX4-K11 site mutation, more HGC-27 cells migrated and invaded, and the difference was statistically significant (Fig. S7C, D).

### CST1 promotes distant metastasis in vivo

**Fig. 7:**
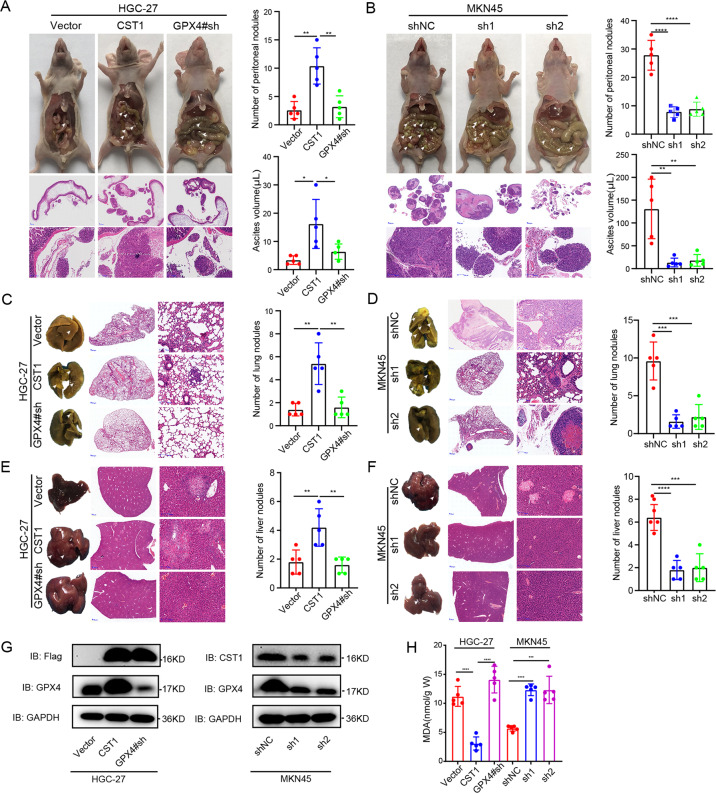
CST1 promotes distant metastasis in vivo. **A** Left: Images of peritoneal metastasis in nude BALB/c mice after injection of HGC-27-Vector/HGC-27-CST1/HGC-27-GPX4#sh cells into their abdominal cavity; images taken 60 days after injection. Right: statistical significance of the peritoneal nodules number and the ascites volume assessed by paired *t*-test, *p < 0.05, **p < 0.01. **B** Left: CST1-silenced MKN45 peritoneum xenograft tumors 30 days after injection. Right: statistical significance of the peritoneal nodules number and the ascites volume assessed by paired *t*-test, **p < 0.01, ****p < 0.0001. **C** Left: corresponding images of the lungs after injection of HGC-27-Vector/HGC-27-CST1/HGC-27-GPX4#sh cells by tail vein; images taken 3 months after injection. Right: statistical significance of the metastasis nodules number assessed by paired *t*-test, **p < 0.01. **D** Left: lung metastasis model of MKN45-shNC/MKN45-sh1-CST1/MKN45-sh2-CST1 8 weeks after tail vain injection. Right: statistical significance of the metastasis nodules number assessed by paired *t*-test, ***p < 0.001. **E**, **F** Left: HGC-27-Vector/HGC-27-CST1 and MKN45-shNC/MKN45-sh1-CST1/MKN45-sh2-CST1 cells were injected into tail vein; metastatic tumors in the livers were assessed 2 and 3 months after injection. Right: statistical significance of the metastasis nodules number assessed by paired *t*-test, **p < 0.01, ***p < 0.001. **G** Western blot analysis indicated metastasis tumors for GPX4 and CST1. **H** The content of MDA in the peritoneal metastatic tumor tissue of nude mice was detected, and the difference was statistically significant (***p < 0.001, ****p < 0.0001). HE-stained sections were magnified ×0.66 and ×5, respectively.

To validate the biological function of CST1 in GC metastasis in vivo, we injected CST1-overexpressing cells (HGC-27-CST1), their corresponding controls (HGC-27-Vector), and HGC-27-GPX4#sh cells into the abdominal cavity of nude mice. After 60 days, metastasis in the peritoneum was analyzed. As expected, ectopic expression of CST1 significantly increased the number of peritoneum xenograft tumors and the ascites volume in the abdominal cavity. However, sh-GPX4 effectively rescued the role of CST1 in the peritoneum metastasis model (Fig. 7A). Conversely, CST1-silenced MKN45 peritoneum xenograft tumors and ascites volume in the abdominal cavity were lower than in control groups (Fig. 7B).

To further explore whether CST1 could promote GC cell metastasis in vivo, HGC-27-Vector/HGC-27-CST1/HGC-27-GPX4#sh were injected into the lateral tail veins of nude mice. Metastasis formation was measured by continuous pathological sections and HE staining 3 months after cell injection. The number of lung metastasis tumors and liver metastasis was significantly increased in the CST1 overexpression group compared with the control group, and the GPX4#sh group could rescue the role of the CST1 (Fig. 7C, E). Contrary, mice injected with MKN45-sh1-CST1/MKN45-sh2-CST1 showed less lung metastasis and liver metastasis compared to control (Fig. 7D, F).

Finally, the role of CST1 was further examined in ex vivo. Immunoblot showed that in tumor grinding cells of HGC-27-Vector/HGC-27-CST1/HGC-27-GPX4#sh, CST1 overexpression increased the expression of the GPX4. The knockdown of CST1 had the opposite effect (Fig. 7G). In addition, the expression of MDA in tumor tissue decreased after overexpression of CST1, while it increased again after the downregulation of GPX4; opposite effects were seen after down-regulation of CST1 (Fig. 7H).

To sum up, this data suggests that CST1 can promote gastric cancer metastasis in vivo through GPX4 inhibition of ferroptosis.

### High levels of CST1 and GPX4 expression correlate with tumor aggressiveness and poor clinical outcome in GC patients

**Fig. 8:**
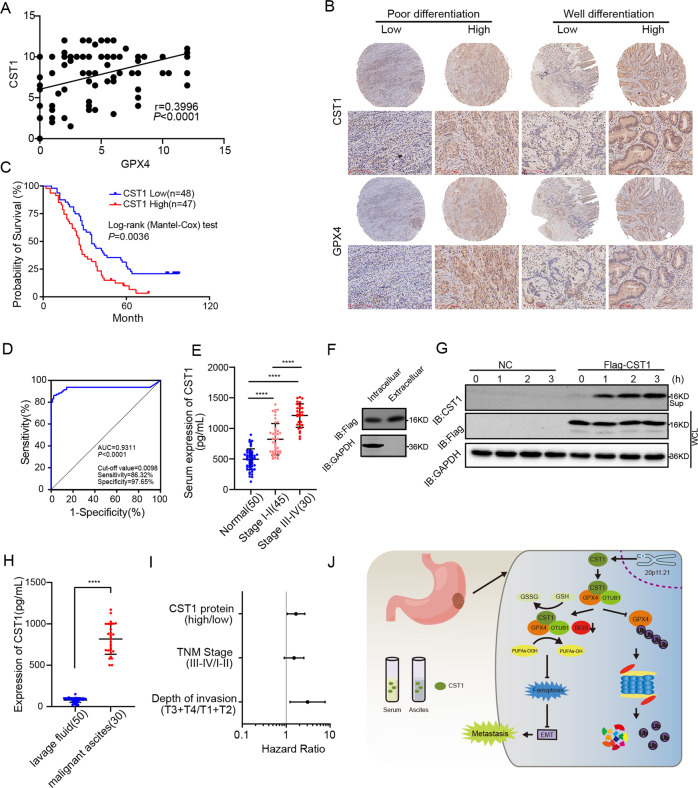
High CST1 and GPX4 expression levels correlate with tumor aggressiveness and poor clinical outcome in GC patients. **A** IHC for 95 GC patients’ tissue. Pearson correlation analysis of the expression of CST1 and GPX4. **B** Representative graph of CST1 and GPX4 expression in IHC according to the degree of differentiation of gastric cancer tissues. **C** Survival analysis; the survival time of 95 gastric cancer patients was analyzed; patients with high CST1 expression had a shorter survival (*p* = 0.0036). **D** ROC curve analysis of the sensitivity and specificity of CST1 in the diagnosis of gastric cancer patients (AUC = 0.9311, *p* < 0.0001). **E** Serum ELISA assay in 50 normal people, 45 stage I–II GC patients, and 30 stage III–IV GC patients revealed that CST1 was highly expressed in GC patients’ serum compared with normal people and highly expressed consistent with patient’s clinicopathological stage (*p* < 0.0001). **F**, **G** Western blot detecting intracellular and extracellular CST1 in HEK293T cells with the FLAG-CST1 expression vector. **H** Peritoneal lavage fluid of 50 GC patients without metastasis and 30 GC patients with malignant ascites by ELISA. Expression of CST1 in malignant ascites was significantly higher than without metastasis patients (*p* < 0.0001). **I** Multivariate Cox regression model analysis of the relationship between CST1 expression and prognosis of gastric cancer. **J** Mechanistic diagram showing the role of CST1 in gastric cancer cells.

To further define the role of CST1 and GPX4 in GC patients, we evaluated their expression in 95 GC patients’ tissue by IHC. Statistical analysis revealed a significant positive correlation when the expression level of CST1 in 95 tissues was plotted against that of GPX4 (*p* < 0.0001) (Fig. 8A). Also, the positive correlation between CST1 and GPX4 expression levels remained unchanged regardless of the degree of gastric cancer tissue differentiation (Fig. 8B). These findings suggested that CST1 and GPX4 might be coregulated in GC.

We performed WB detection of GPX4 protein in 5 paired gastric cancer tissues and adjacent normal gastric mucosa tissue samples, the results showed that GPX4 protein levels were higher in gastric cancer tumor tissues than in adjacent normal gastric mucosa tissues (Fig. S8A), which is in good agreement with the expression of CST1 in Fig. 1L. At the same time, we detected the expression of GPX4 protein in normal gastric mucosa epithelial cells GES-1 and gastric cancer cell lines AGS etc., the results showed that the level of GPX4 protein in gastric cancer cells increased (Fig. S8B), which was a good correlation with the expression of CST1 protein in Fig. 2B.

In order to explore whether the expressions of CST1 and GPX4 are also related in other tumor tissues, we selected colon cancer tissues and cells for experiments. We performed WB detection of CST1 and GPX4 proteins in 5 matched colon cancer tissues and adjacent normal intestinal mucosal tissue samples. The results showed that compared with adjacent normal intestinal mucosal tissues, the levels of CST1 and GPX4 proteins in colon cancer tumor tissues were higher (Fig. S8C), and the expression of the two molecules were correlated. At the same time, we detected the expression of CST1 and GPX4 proteins in normal intestinal mucosal epithelial cells HIEC and colon cancer cell lines HCT116 etc., the results showed that the protein levels of CST1 and GPX4 were significantly increased in colon cancer cells, while their expression was lower in HIEC. And the expression of the two molecules showed a good correlation in colon cancer cells (Fig. S8D).

The clinical and pathological features are described in Table S2; information on the cohort included age, gender, tumor size, differentiation, TNM stage, and follow-up time. The patients were followed for 1–112 months (median follow-up time was 60 months). Next, we determined the potential clinicopathologic implications of altered CST1 expression. The CST1 levels were significantly higher in patients with poor overall survival (*p* = 0.0036, Fig. 8C), poor differentiation (*p* = 0.027, Table S2), and having lymph node invasion (*p* = 0.039, Table S2). Moreover, CST1 improved the sensitivity and specificity of gastric cancer diagnosis (Fig. 8D).

Moreover, CST1 was highly expressed in the serum of patients with GC. Serum ELISA assay in 50 normal people, 45 stage I-II GC patients, and 30 stage III-IV GC patients revealed that CST1 was highly expressed in GC patients’ serum compared with normal people and highly consistent with patient’s clinicopathological stage (*p* < 0.0001, Fig. 8E). To further define the role of CST1 in GC patients, we constructed a FLAG-CST1 expression vector, which was transfected in HEK293T cells. Western blot revealed that CST1 was expressed intracellularly and extracellularly, and with the prolongation of time, the extracellular gradient expression of CST1 increased (Fig. 8F, G).

Finally, we conformed peritoneal lavage fluid of 50 GC patients without metastasis and 30 GC patients with malignant ascites by ELISA. Expression of CST1 in malignant ascites was significantly higher than in patients without metastasis (*p* < 0.0001, Fig. 8H). Univariate and multivariate Cox regression model analysis revealed that high expression of CST1 was an independent risk factor for the prognosis of gastric cancer patients (Fig. 8I and Table S3).

These data demonstrated that CST1 and GPX4 participate in the malignant progression of GC, and serological detection of CST1 in GC patients provides a strong diagnostic basis for the diagnosis, recurrence, and metastasis detection of GC patients.

## Discussion

In the present study, we identified CST1 as one of the regulators promoting gastric cancer metastasis. Mechanistically, CST1 relieves GPX4 ubiquitination by recruiting the deubiquitinating enzyme OTUB1, improving GPX4 protein stability and reducing intracellular reactive oxygen species ROS, thereby inhibiting ferroptosis and, in turn, promoting EMT and metastasis of gastric cancer cells (Fig. 8J).

The roles of CST1 in the metastasis of different cancers have already been reported in the literature. Cui et al. discovered that CST1 could modulate the EMT through the PI3K/AKT pathway, thereby promoting the malignant progression of hepatocellular carcinoma [32]. Ding and colleagues suggested that CST1 regulates papillary thyroid carcinoma cells invasion, migration, and EMT [31]. Although some studies have shown that CST1 promotes the malignant progression of gastric cancer, the specific mechanism of CST1 affecting the migration, invasion, and metastasis of gastric cancer remain poorly understood, especially mechanisms on the relationship between CST1 and EMT of gastric cancer.

In this study, we performed whole transcriptome sequencing and bioinformatic analysis and found that the prognosis of gastric cancer patients with high CST1 expression was poor. Subsequently, in vitro and in vivo experiments indicated that the overexpression of CST1 promoted the migration, invasion, and metastasis of gastric cancer cells in nude mice, but had no effect on cell proliferation.

Then, we used MKN45 cells to discover 63 proteins that interact with CST1 through co-immunoprecipitation and mass spectrometry. Through KEGG pathway analysis, we were surprised to find that the CST1 interacting proteins were mainly involved in the regulation of ferroptosis. Furthermore, we verified the proteins involved in ferroptosis in vitro and found that CST1 binds to GPX4. Our results revealed that after up-regulating the expression of CST1, the ferroptosis inducer erastin-induced cell death decreased and the level of ROS, MDA decreased, and GSH increased; while after down-regulating the expression of CST1, erastin-induced cell death increased, and at the same time, the level of ROS, MDA increased, and GSH decreased. The above results indicate that CST1 inhibits the occurrence of ferroptosis through GPX4.

Experimental results showed that GPX4 mRNA did not significantly change after the expression of CST1 was up-regulated or down-regulated, but the expression of GPX4 protein changed, suggesting that CST1 may stabilize the protein level of GPX4 through post-translational modification. The degradation of GPX4 protein was mainly mediated by UPS and autophagy. In the lung cancer cell line A549, the deubiquitinase inhibitor PdPT increases the ubiquitination of GPX4 and promotes the protein degradation of GPX4 [33]. In order to explore which pathway CST1 affects the expression of GPX4, we treated cells with a protein synthesis inhibitor CHX. In cells overexpressing CST1, the protein degradation of GPX4 was significantly reduced, suggesting that CST1 affects GPX4 through the ubiquitin-proteasome system stability of a protein. Further application of the inhibitor MG132 to the cells up-regulated by CST1 revealed that the ubiquitination modification of GPX4 was significantly reduced. Therefore, we speculated that CST1 stabilizes the GPX4 protein by reducing the ubiquitination modification of the GPX4 protein, maintains the homeostasis of intracellular reactive oxygen species, and protects the cells against ferroptosis.

The deubiquitinating enzyme (DUB) participates in the deubiquitination modification of the target protein. The deubiquitinating enzyme OTUB1 enhances the sensitivity of tumor cells to erastin-induced ferroptosis by stabilizing the proteasome-dependent SLC7A11 [27]. The ubiquitin ligase NEDD4L mediates the degradation of lactoferrin LTF, reduces the transport of iron ions, and reduces oxidative damage [34]. However, the deubiquitinase and ubiquitin ligase for GPX4 have not yet been reported. We speculate that CST1 resists ferroptosis by recruiting DUB to relieve the ubiquitination modification of GPX4, or that CST1 affects the activity of ubiquitin ligase, and the mechanism needs to be further explored.

Through bioinformatic analysis of our previous transcriptome sequencing data and GEO data set, we found that gastric cancer tissues highly expressed CST1 and enriched the deubiquitination pathway. By further predicting the deubiquitinase DUB that interacts with GPX4 through the online database, we found that the proteins that may potentially interact with GPX4 include OTUB1 and OTUD5. Co-IP experiments confirmed that CST1 could bind to OTUB1, and OTUB1 regulated the ubiquitination of GPX4 protein. We demonstrated that CST1 relieves the ubiquitination of GPX4 through OTUB1, thereby promoting ferroptosis resistance in gastric cancer cells.

Deubiquitinating enzymes belong to the proteasome superfamily. They reversely regulate protein degradation by hydrolyzing the links between ubiquitin chains of substrate proteins, thereby affecting or regulating cell metabolism, differentiation, and proliferation [35]. Recent studies have found that deubiquitinases can selectively regulate cancer-related proteins, which are closely related to the occurrence and development of tumors [36]. Ovarian tumor-associated protease B1 (OTU domain-containing ubiquitin aldehyde-binding protein B1, OTUB1) is a member of the DUB family widely expressed in the kidneys, intestine, brain, liver, and lungs [37]. OTUB1 is a non-canonical deubiquitinase involved in the malignant progression of multiple tumors [38]. OTUB1 promotes colorectal cancer metastasis by facilitating EMT and acts as a potential distant metastasis marker and prognostic factor in CRC [39]. OTUB1 also facilitates metastasis of esophageal squamous cell carcinoma (ESCC) by promoting snail protein stability [40]. In addition, OTUB1 is a deubiquitinating enzyme that influences cancer immunosuppression via regulation of PD-L1 stability and a potential therapeutic target for cancer immunotherapy [29]. To the best of our knowledge, this is the first study that revealed how CST1 can recruit OTUB1 to stabilize GPX4 protein, thereby inhibiting ferroptosis and promoting gastric cancer metastasis.

Furthermore, we found that CST1 promotes EMT in gastric cancer. In addition to genetic or epigenetic changes, tumor cells require the participation of various stimuli in the tumor microenvironment, including the release of cytokines, growth factors, and metabolic changes. In the remodeling of the extracellular matrix, reactive oxygen species (ROS) have a “double-edged sword” role [41]. Studies have reported that an increase in ROS accompanies the induction of EMT in cancer cells; yet, excessive ROS can also cause tumor cell death [42]. Previous studies have reported that cells in the interstitial state are more sensitive to ferroptosis-inducing agents during EMT [43]. These studies suggest that a large amount of ROS is produced during the occurrence of EMT, making cells sensitive to ferroptosis inducers. Our research shows that CST1 reduces intracellular ROS by stabilizing GPX4 protein, thereby maintaining the ROS homeostasis of gastric cancer cells in the EMT state, inhibiting ferroptosis and, in turn, promoting the metastasis of gastric cancer.

We also found that the expression of CST1 was elevated in specimens of gastric cancer, which is closely related to the degree of differentiation of gastric cancer tissue and lymph node metastasis. Compared with the peripheral blood test results of healthy controls, the expression of circulating CST1 in patients with gastric cancer was increased and was closely related to disease progression. Furthermore, ROC curve analysis indicated that CST1 improved the sensitivity and specificity of the diagnosis of gastric cancer patients. Compared with the peritoneal lavage fluid of patients with early gastric cancer and advanced gastric cancer, we detected a significant increase in the expression of CST1 in the ascites of gastric cancer patients with peritoneal metastasis, which suggested that CST1 was involved in the peritoneal metastasis of gastric cancer and was closely related to the prognosis and survival of patients.

In summary, in the process of gastric cancer metastasis, the expression of CST1 increases. By recruiting OTUB1, which binds to GPX4, CST1 inhibits the ubiquitination and degradation of GPX4 protein, promotes protein stability, reduces intracellular reactive oxygen species, and protects cells against ferroptosis. At the same time, ROS reduces the remodeling of the cell microenvironment and promotes the EMT and metastasis of gastric cancer. Also, the detection of patients’ peripheral blood and ascites samples could be helpful for gastric cancer diagnosis, malignant progression and prognosis evaluation.

## Materials and methods

### Tissue specimens

Primary GC tissues and the corresponding non-cancerous adjacent tissues were collected from 185 patients who underwent gastric resection for GC without neoadjuvant therapy at the First Affiliated Hospital of Soochow University from 2009 and 2019. Ninety-five GC patients completed follow-up and underwent clinicopathological and prognostic analysis. Among them, 4 pairs of primary GC and adjacent normal tissues without metastasis, and 4 pairs of primary GC and adjacent normal tissues with peritoneal metastasis were used for whole transcriptome sequencing (OE Biotech, Shanghai, China). The remaining samples were stored at −80 °C for RT-qPCR or were fixed in 10% formalin and embedded in paraffin for IHC. Peripheral blood samples, malignant ascites, or peritoneal lavage from these GC patients and healthy donors were collected for ELISA.

All patients provided written informed consent. This study was performed in full accordance with the principles of the Declaration of Helsinki. The Ethics Committee of the First Affiliated Hospital of Soochow University approved this study (approval number: 2020381).

### Cell lines and cell culture

Human GC cell lines AGS, HGC-27, MKN45, SNU-1 were purchased from the Procell Life Science & Technology Co., Ltd. (Wuhan, China); MGC803 were purchased from the Beyotime Biotechnology Company (Shanghai, China). HEK293T, normal human gastric mucosal epithelial cell line GES-1, human GC cell lines BGC-823, SGC7901, and the human colon cancer cell lines HCT116, HCT-8, SW480, KM12 and normal human intestinal mucosal epithelial cell line HIEC were all stored in Dr. Zhou’s laboratory under standard conditions. HGC-27 and MKN45 were recently authenticated using standard short tandem-repeat-based DNA profiling (STR) (Supplementary file 1). All cell lines were free from mycoplasma contamination. Cells were cultured in RPMI 1640, or DMEM medium, supplemented with 10% fetal bovine serum (FBS) (Procell, Wuhan, China) and 1%Penicillin/Streptomycin in a humidified atmosphere containing 5%CO_2_/95% air at 37 °C.

### Quantitative real-time PCR (RT-qPCR)

Total RNA was isolated from GC tissues or cultured cells using a Trizol (Invitrogen) standard protocol. The integrity, quantity, and purity of RNA were examined using NanoDrop 2000c Spectrophotometer (Thermo Scientific, Wilmington, USA). Briefly, total RNA (1 µg) was reverse transcribed using All-In-One 5 × RT MasterMix (ABM, Canada). Real-time quantitative PCR reactions were then performed on an ABI ViiA7 Sequence Detection System (Life Technologies, USA) using SYBR Green Master Mix (ABI). Relative gene expression levels were analyzed using comparative Ct methods where Ct was the cycle threshold number normalized to GAPDH. The primers are shown in Table S1.

### Western blot (WB)

Cell lysates were prepared in radioimmunoprecipitation assay (RIPA) buffer supplemented with a proteinase inhibitor. Protein concentrations were quantified with the BCA Protein Assay Kit (Beyotime Biotechnology, China). Proteins (20 μg) were separated by SDS-PAGE and electroblotted onto PVDF membranes. Membranes were then blocked in TBST containing 5% BSA for an hour and then incubated with primary antibody at 4 °C overnight. After washing 3 times with TBST (10 min for each time), membranes were incubated with secondary antibodies diluted in blocking buffer for 1 h at room temperature. Samples were then washed again with TBST three times, after which an enhanced chemiluminescence (ECL) was performed using an ECL kit (Beyotime Biotechnology, Shanghai, China). Densitometric analysis of each band was measured using ImageJ software for quantification.

The antibodies against GPX4 (rabbit monoclonal, Cat# ab125066, used at 1:5000), OTUB1 (rabbit monoclonal, Cat# ab175200, used at 1:1000), GAPDH (Mouse monoclonal, Cat# ab8245, used at 1:1000) were purchased from Abcam (Cambridge, MA). CST1 antibody (rabbit polyclonal, Cat# 16025-1-AP, used at 1:1000) was purchased from Proteintech (Wuhan, China). Flag antibody (rabbit polyclonal, Cat# 20543-1-AP, used at 1:1000) was purchased from Proteintech (Wuhan, China). C-Myc antibody (rabbit polyclonal, Cat# 10828-1-AP, used at 1:1000) was obtained from Proteintech (Wuhan, China). HA-tag antibody (rabbit polyclonal, Cat#51064-2-AP, used at 1:1000) was purchased from Proteintech (Wuhan, China). HRP-conjugated goat-anti-mouse or rabbit antibody (used at 1:2000) was acquired from Epizyme Biomedical Technology Co., Ltd (Shanghai, China).

### Colony formation assay

Cell proliferation ability was measured by plate colony formation assay. Briefly, 500 cells were added to each well of a 6-well plate and incubated for ~2 weeks until a colony was obviously formed; the medium was regularly changed. Next, the plate was gently washed and stained with 0.1% crystal violet, and the number of colonies was counted.

### CCK8 assay

CCK8 assays were performed according to the manufacturer’s manual (Beyotime Biotechnology, Shanghai, China). Briefly, 2000 cells in 100 μl culture were added into each well of a 96-well plate for 24, 48, and 72 h. At each time point, 10 μl of sterile CCK-8 was added to each well and incubated for another 2 h at 37 °C. The absorbance at 450 nm was determined using a microplate reader.

### Cell migration and invasion assay

A wound-healing assay was used to test cell migration ability. Briefly, cells in the exponential phase of growth were harvested and seeded in a 6-well plate. After the cell reached 90% confluence, a line was drawn using a marker on the bottom of the dish, after which a sterile 100 μl pipet tip was used to scratch three separate wounds through the cells, moving perpendicular to the line. Next, the cells were gently rinsed twice with PBS to remove floating cells. Images of the scratches were taken using an inverted microscope at ×10 magnification at 0 and 24 h of incubation.

Cell invasion assays were performed in 24-well plates with 8.0 mm pore inserts pre-coated with Matrigel (BD, 356234). Briefly, GC cells were digested and resuspended in FBS-free 1640 culture medium. A total of 200 μl (5 × 10^5^cells/ml) of GC cells was seeded into the upper chamber, while a 600 μl complete culture medium (1640 with 10% FBS) was added to the lower chamber. Cells were incubated for 24 h, 48 h, and 72 h. For each insert, the invading cells in five random fields of ×400 magnification were counted. Each experiment was performed in triplicate, and the mean values were shown.

For the migration assays, a total of 5 × 10^4^ GC cells were seeded into 8.0 mm pore inserts without Matrigel. The migration time was 12 h.

### Small interfering RNAs (siRNAs), plasmid and lentivirus construction, and transfection protocol

All siRNAs, Flag-tagged OTUB1, Flag-tagged OTUD5, Myc-tagged CST1, and HA-tagged GPX4 plasmids were purchased from Vigene Biosciences (Shandong, China). All plasmids were generated by cloning the corresponding cDNA into the expression vector pCMV-MCS at the AsisI and M1uI restriction sites. D88A mutant plasmid of OTUB1 and K11A mutant plasmid of GPX4 were constructed in our laboratory by using QuickMutation™ Site-Directed Mutagenesis Kit (Beyotime Biotechnology, Shanghai, China). HEK293T or GC cells were transfected with siRNAs and/or plasmids using Lipofectamine 3000 (Invitrogen, CA, USA) according to the manufacturer’s instructions. To establish a stable cell line, the pLenti6.3/IRES/GFP lentiviral plasmid carrying green fluorescent protein (GFP) and puromycin resistance genes were used for the preparation of CST1 overexpression recombinant lentivirus. pLKO.1-puro lentiviral plasmid was used to prepare CST1 interference lentivirus. After 72 h of infection, 10 μg/ml puromycin was used for screening cells overexpressing CST1 and those with CST1 knockdown. Fluorescence microscope and flow cytometry were used to detect GFP and analyze the efficiency of lentivirus-mediated transgenesis. RT-qPCR and Western blot were used to detect overexpression and silencing efficiency of lentivirus-mediated CST1 in GC cells.

### Immunohistochemistry (IHC)

5μm-thick paraffin-embedded sections of clinical specimens and mice metastatic samples were used for IHC staining following the previously reported methods. The final score was taken as the median: < the median indicated low expression, while > the median indicated a high expression. The IHC sections were further scanned and analyzed respectively with NanoZoomer S60 (Hamamatsu Photonics) and ImageJ.

### Co-IP and LC-MS/MS

Total proteins were extracted from the inflorescence tissues with IP buffer (50 mM Tris-HCl, pH 7.6, 150 mM NaCl, 5 mM MgCl_2_, 10% glycerol, 0.1% NP-40, 0.5 mM DTT, and protease inhibitor cocktail), and then precipitated with anti-Flag (Sigma-Aldrich) or anti-Myc (Millipore) antibodies for 2 h at 4 °C. After five times washing, the precipitated protein mixtures were subjected to LC-MS/MS analysis.

### Determination of intracellular ROS, malondialdehyde (MDA), and glutathione (GSH)

Intracellular ROS level was detected by flow cytometry and fluorescence microscopy with CellROX™ Deep Red Reagent (Invitrogen) as the protocol. Briefly, samples were incubated in 5 μM reagent before treatment for 30 min. Then, 0.5 h after treatment, cells were collected for fluorescence intensity detection. Malondialdehyde (MDA) detection Kit and GSH detection Kit (Solarbio Science & Technology Co. Ltd., Beijing, China) were used to analyze the production of MDA and GSH in GC cells and tumor tissues.

### In vivo metastasis assay

Male BALB/c nude mice, 4–5 weeks, were obtained from the Shanghai SLAC Laboratory Animal Co., Ltd. Animals were kept in a specific pathogen-free environment with a temperature of 22 ± 1 °C, relative humidity of 50 ± 1%, and a light/dark cycle of 12/12 h and given water and food ad libitum. All animal studies (including the mice euthanasia procedure) were done in compliance with Soochow University institutional animal care regulations and conducted according to the AAALAC and the IACUC guidelines (approval number: 202109A0101).

When establishing a peritoneal metastasis model, 1 × 10^7^ HGC-27-Vector/HGC-27-CST1 cells in 200 µl sterile PBS mixed with matrigel were injected into the nude mice abdominal cavity for 60 days (*n* = 5 per group). In addition, 5 × 10^6^ MKN45-shNC/MKN45-sh1-CST1/MKN45-sh2-CST1 cells were injected into the abdominal cavity of nude mice for 30 days (*n* = 5 per group). Mice were then euthanized, and peritoneal metastases and the number of tumor nodules were analyzed and counted. Dissected tissues were embedded and HE stained.

When establishing lung and liver metastasis models, 5 × 10^6^ GC cells resuspended in 100 µl sterile PBS were injected into the tail veins of nude mice. The mice were euthanized on day 60 (MKN45 stable cell lines) and day 90 (HGC-27 stable cell lines) after injection (*n* = 5 per group). The lungs of mice were then dissected and fixed in Bouin’s fluid (G-CLONE, Beijing, China); the liver was fixed in 4% paraformaldehyde solution and further embedded and HE stained to evaluate tumor metastasis.

### Bioinformatic analysis

Bioinformatic analysis was performed based on a combination of R, command line, and web-based bioinformatics tools. The following public databases were searched: the Cancer Genome Atlas (TCGA) database (https://gdac.broadinstitute.org/), Gene Expression Omnibus (GEO) database (https://www.ncbi.nlm.nih.gov/geo/), and Kaplan-Meier Plotter (http://kmplot.com/analysis/). BioGRID (http://thebiogrid.org/), IntAct (https://www.ebi.ac.uk/intact/home) for protein interaction prediction. The results of RNA-seq data analysis are presented in Supplementary File 2 and 3.

### Protein-protein docking

In order to predict the model of the direct binding between the three protein molecules CST1, OTUB1 and GPX4, we first obtained the 3D spatial structures of these proteins through SWISS-MODE and PDB databases, and then based on the computational protein docking method, using Cluspro (https://cluspro.bu.edu/) online tool predicts the most likely complex model for CST1, OTUB1 and GPX4 binding. The interaction surfaces in protein complexes were further analyzed online by PDBePISA (https://www.ebi.ac.uk/msd-srv/prot_int/pistart.html). Finally, conformational mapping and docking region analysis were performed using PYMOL (https://pymol.org/) and LIGPLOT (https://www.ebi.ac.uk/thornton-srv/software/LigPlus/).

### Statistical analysis

SPSS 26.0 software (IBM Corp.) and GraphPad Prism 9 were used for all statistical analyses. Continuous data were presented as means ± standard deviation (SD), and the differences among the experimental groups were analyzed using one-way ANOVA or Student’s *t* test. Frequencies of categorical variables were compared using Pearson’s *χ*^2^ test. The survival curve was generated using the Kaplan-Meier method and compared by log-rank test. *p* < 0.05 was considered statistically significant.

## Supplementary information

Figure S1

Figure S2

Figure S3

Figure S4

Figure S5

Figure S6

Figure S7

Figure S8

Supplementary figure legends

Table S1

Table S2

Table S3

Supplementary file 1-Cell Line STR Identification Reports

Supplementary file 2-PT-vs-PA-heatmap.reorder_cluster_result

Supplementary file 3-MT-vs-MA-heatmap.reorder_cluster_result

Supplementary file 4-LC-MS of Proteins

## Data Availability

Source data and reagents are available from the corresponding author upon reasonable request.
