# Supplementary figures and images for: CST1 inhibits ferroptosis and promotes gastric cancer metastasis by regulating GPX4 protein stability via OTUB1
Source: Oncogene. 2022 Nov 12;42(2):83–98. doi: 10.1038/s41388-022-02537-x (PMC9816059; doi:10.1038/s41388-022-02537-x)

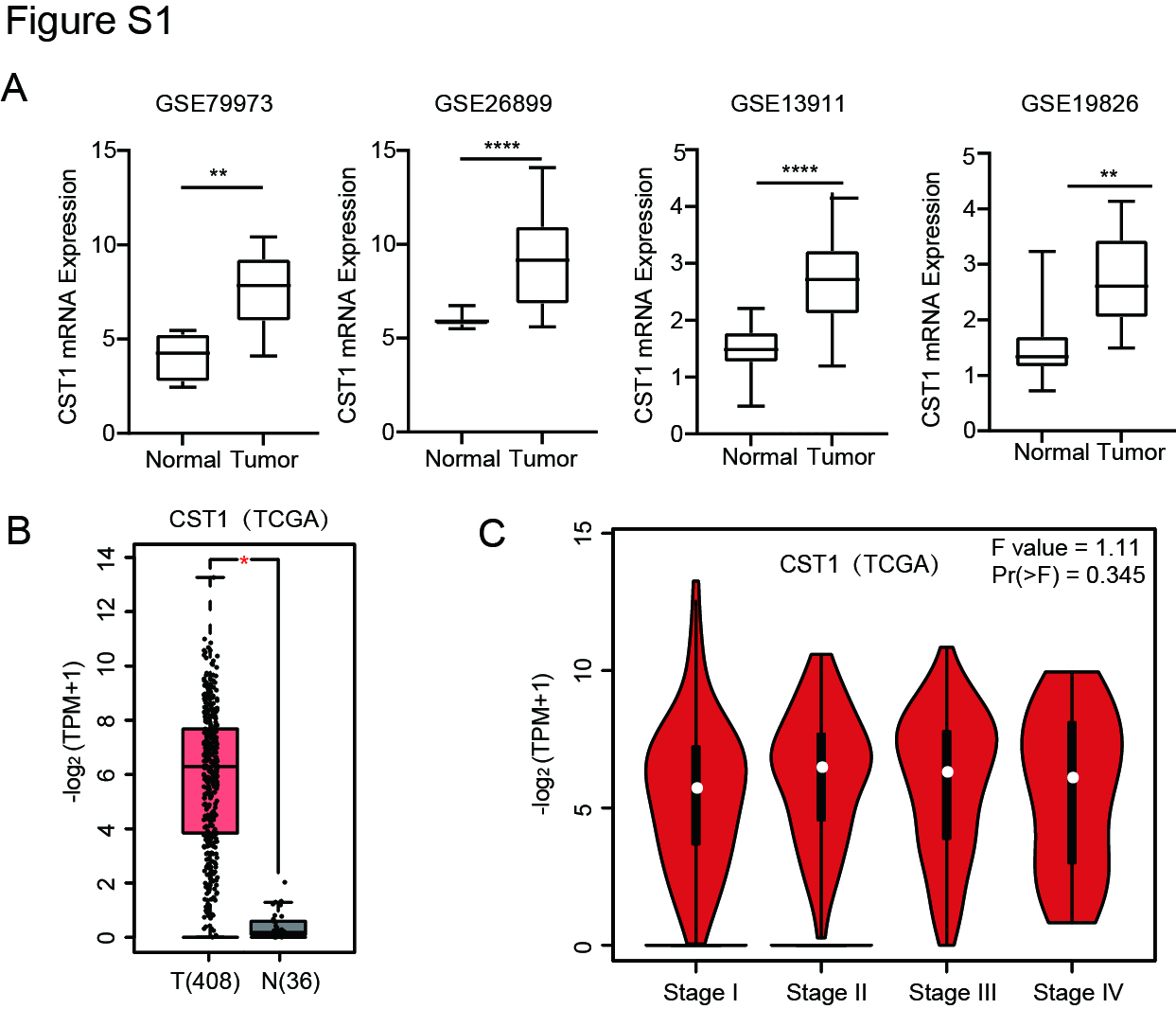

Supplement: Supplementary file 1 — Figure S1 [file 41388_2022_2537_MOESM1_ESM.jpg]

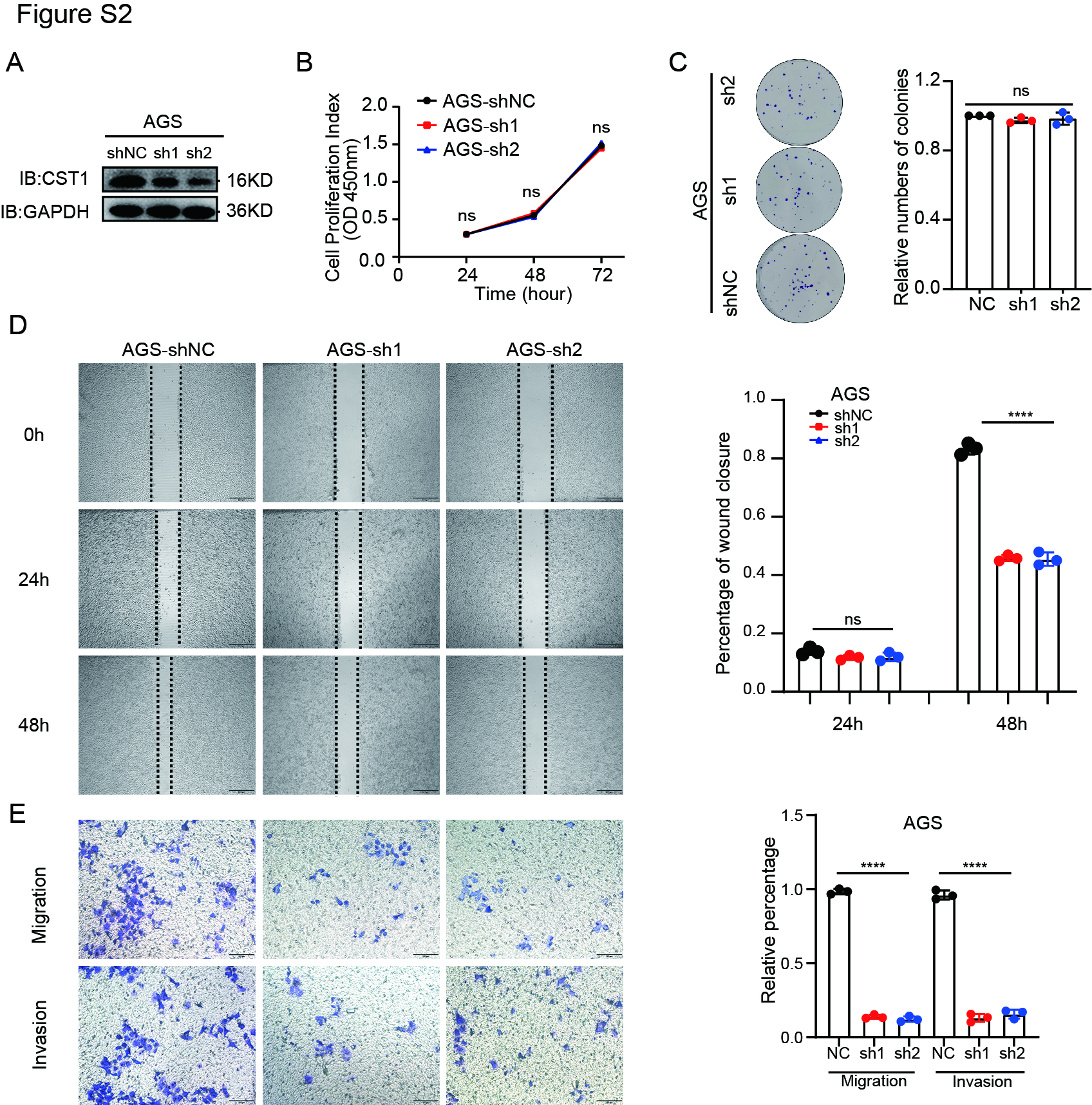

Supplement: Supplementary file 2 — Figure S2 [file 41388_2022_2537_MOESM2_ESM.jpg]

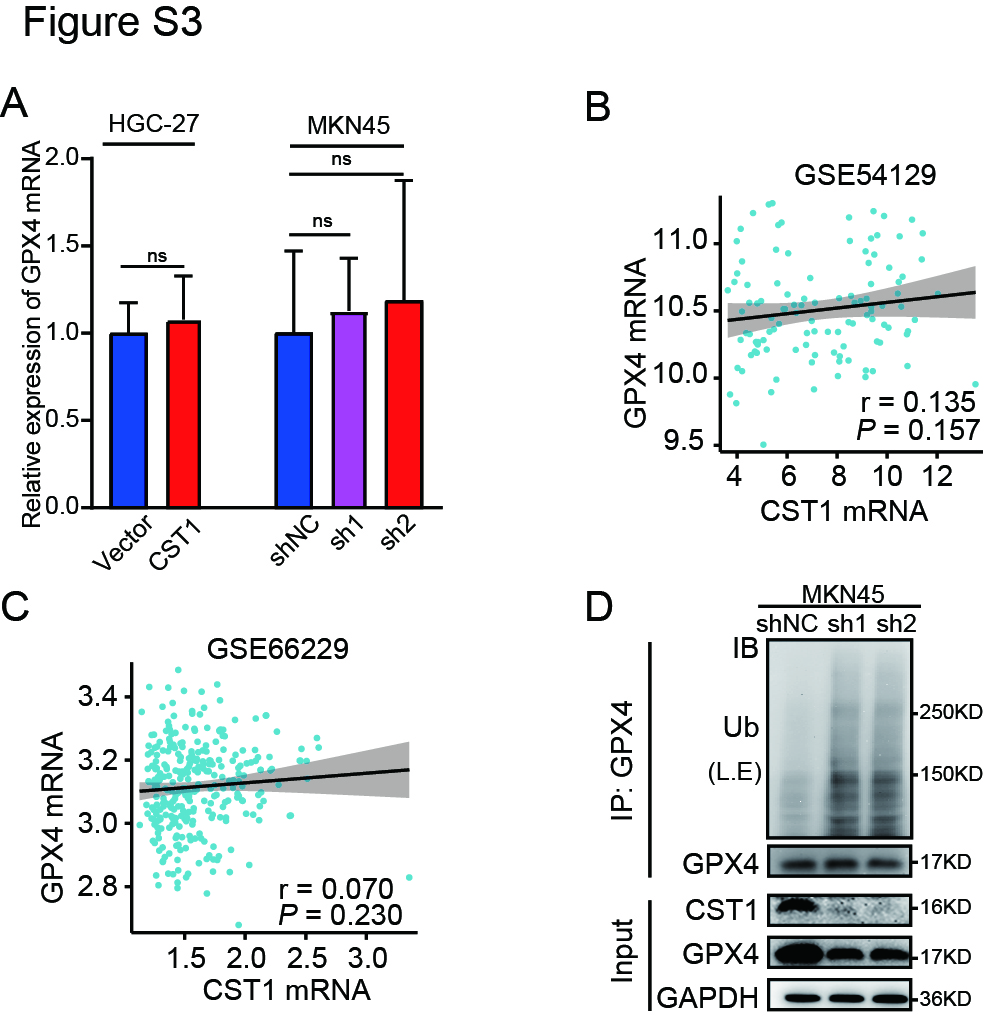

Supplement: Supplementary file 3 — Figure S3 [file 41388_2022_2537_MOESM3_ESM.jpg]

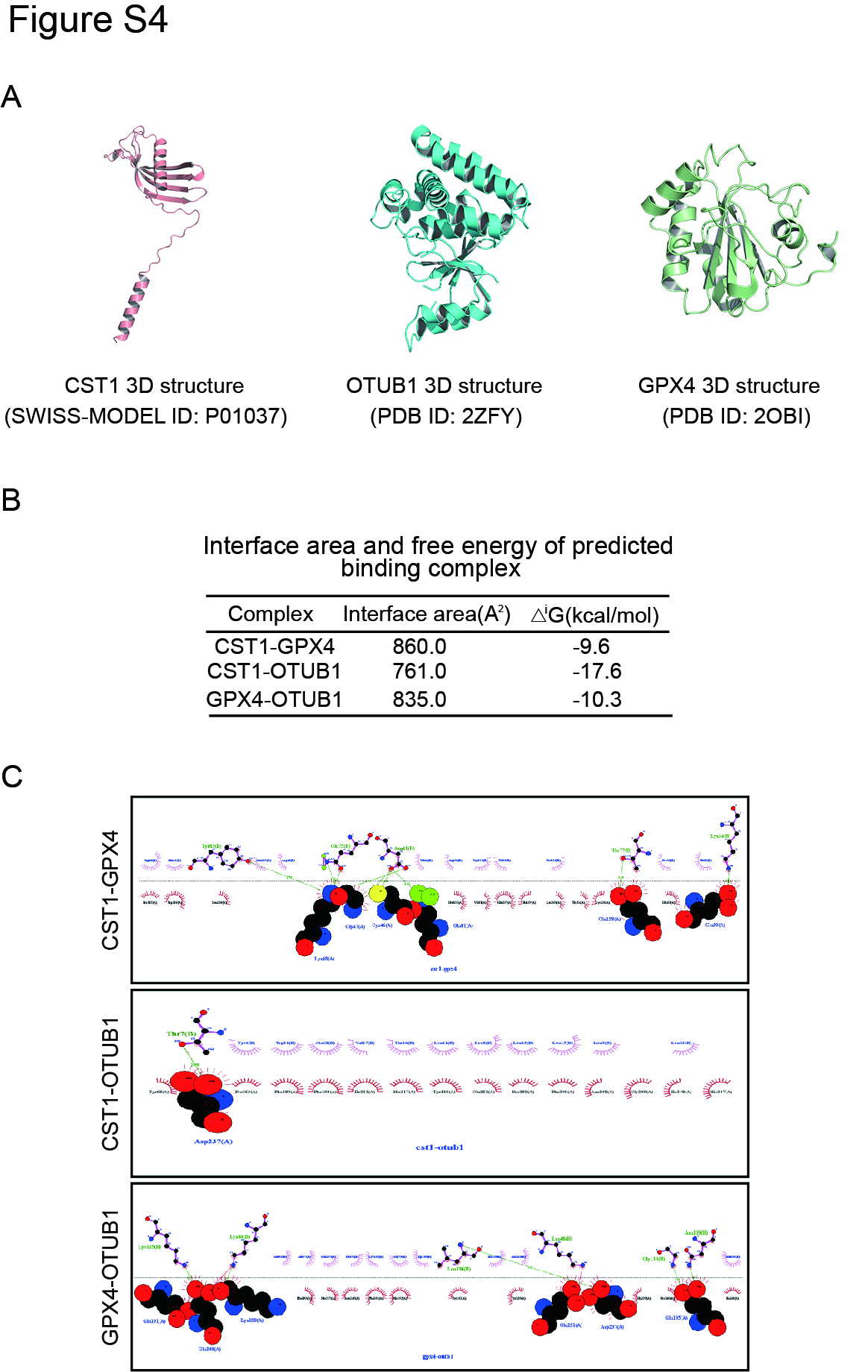

Supplement: Supplementary file 4 — Figure S4 [file 41388_2022_2537_MOESM4_ESM.jpg]

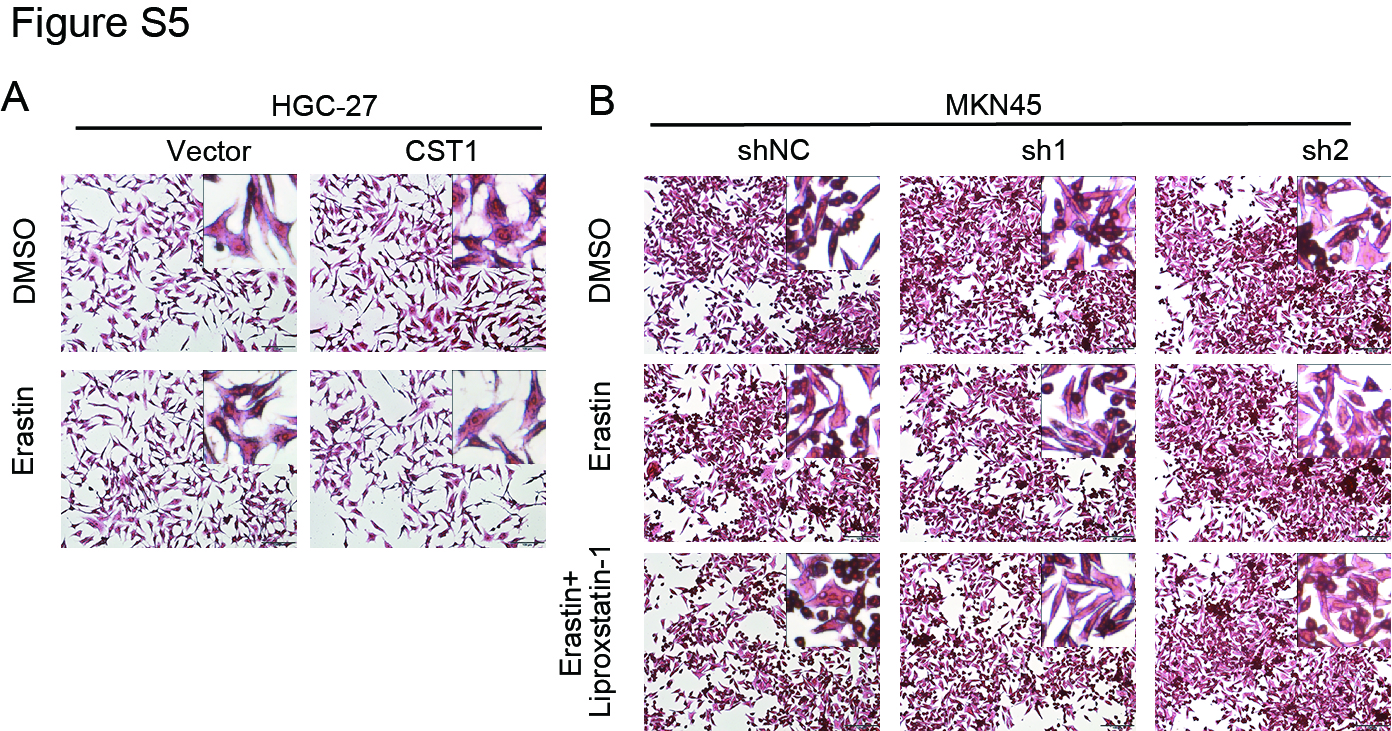

Supplement: Supplementary file 5 — Figure S5 [file 41388_2022_2537_MOESM5_ESM.jpg]

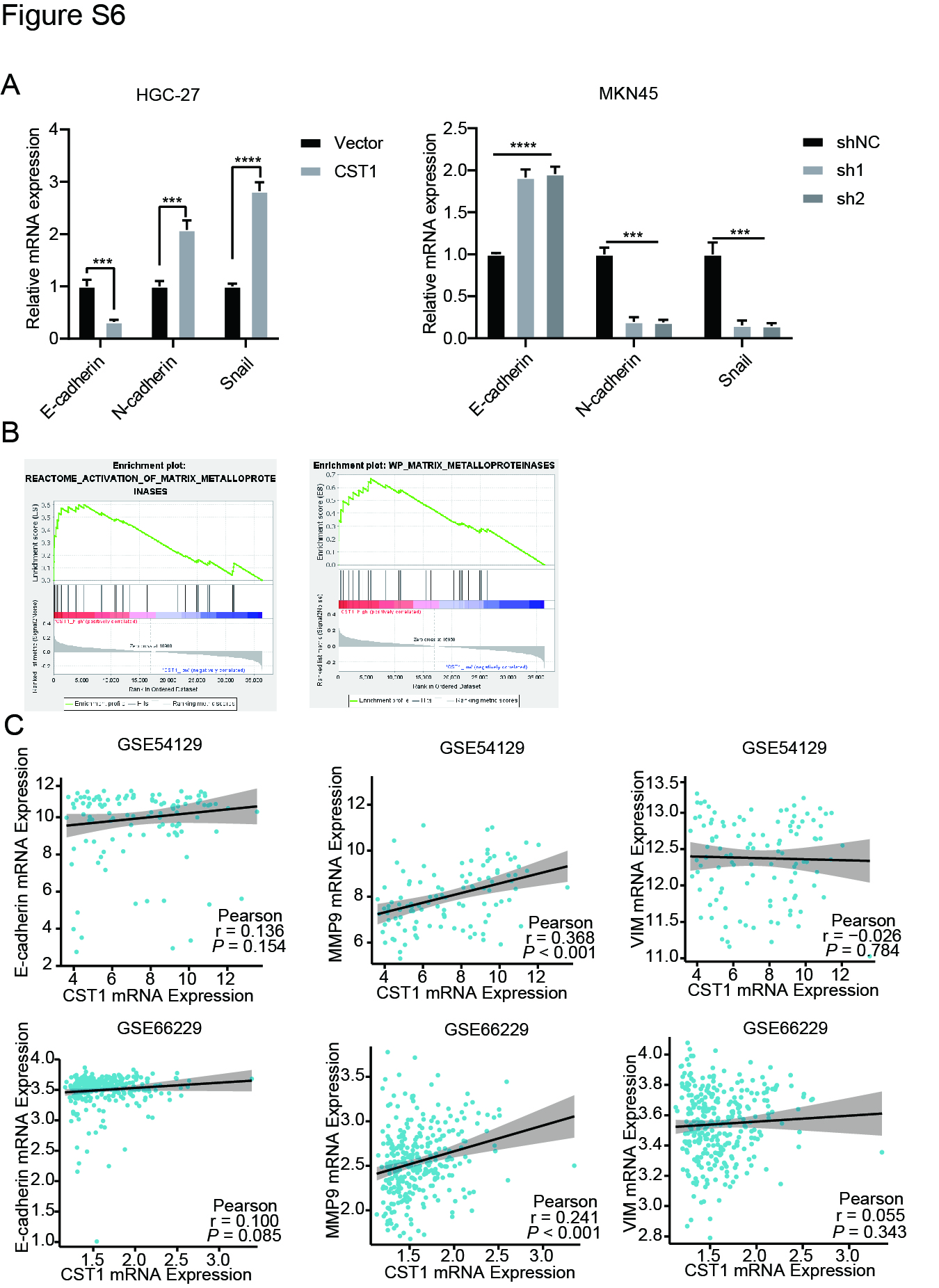

Supplement: Supplementary file 6 — Figure S6 [file 41388_2022_2537_MOESM6_ESM.jpg]

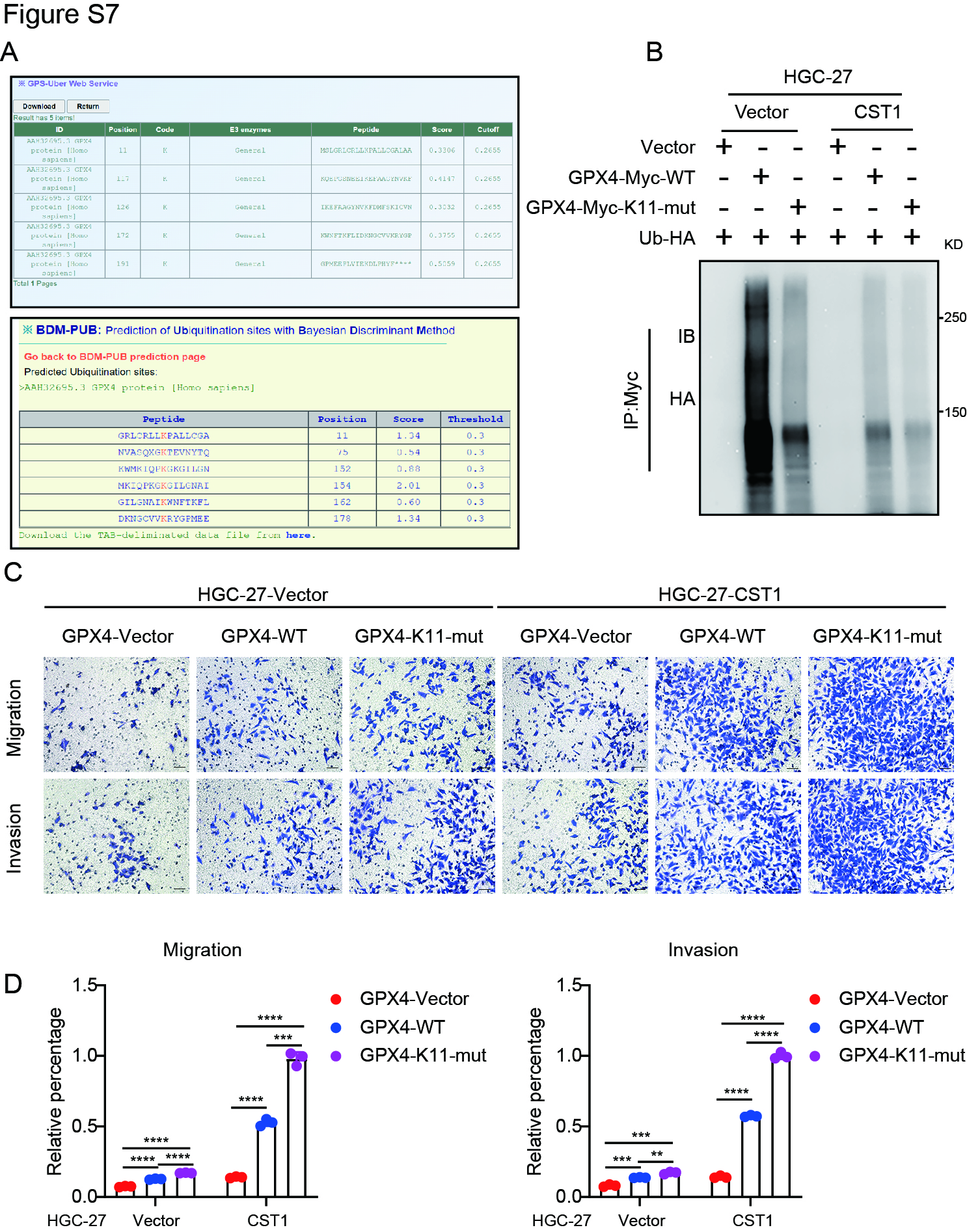

Supplement: Supplementary file 7 — Figure S7 [file 41388_2022_2537_MOESM7_ESM.jpg]

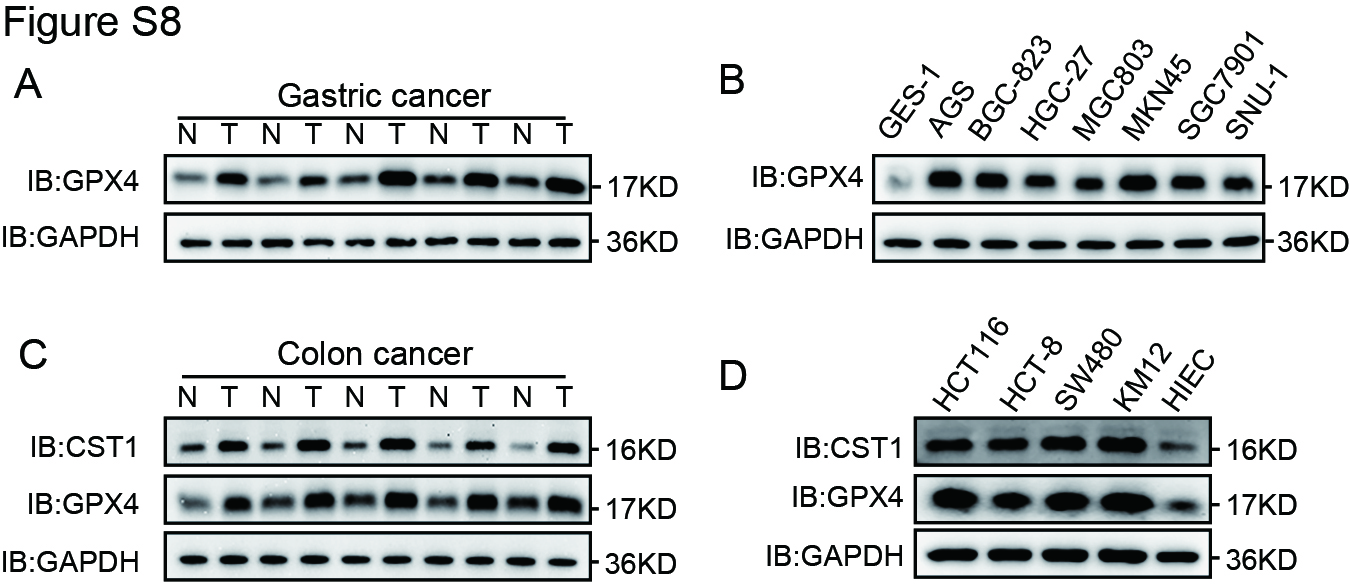

Supplement: Supplementary file 8 — Figure S8 [file 41388_2022_2537_MOESM8_ESM.jpg]
