# Supplementary material for: CST1 inhibits ferroptosis and promotes gastric cancer metastasis by regulating GPX4 protein stability via OTUB1
Source: Oncogene. 2022 Nov 12;42(2):83–98. doi: 10.1038/s41388-022-02537-x (PMC9816059; doi:10.1038/s41388-022-02537-x)
Supplement: Supplementary file 9 — Supplementary figure legends [file 41388_2022_2537_MOESM9_ESM.docx]

**Supplementary figure legends**

**Figure S1. (A)** Higher expression of CST1 was found in GC samples than the matched normal tissues (based on GSE79973, GSE26899, GSE13911 and GSE19826 databases), **<0.01, ****<0.0001. **(B)** Higher expression of CST1 was found in GC samples than the matched normal tissues (based on TCGA database), *<0.05. **(C)** The expression of CST1 in gastric cancer stage II, III and IV was higher than that in stage I, but the difference was not statistically significant (*P*=0.345).

**Figure S2. (A)** Western blot analysis of AGS stably transfected with CST1 knockdown lentiviruses and control lentiviruses. Total GAPDH was used as a loading control. **(B)** CCK8 assay analyzed the proliferation of AGS-shNC/AGS-sh1-CST1/AGS-sh2-CST1 stable cell lines. Data are shown as the mean±SD of triplicate independent sets of experiments; statistical significance was assessed by paired t-test. **(C)** A colony formation assay. Left panel: representative images, right panel: quantification analysis. Data from independent experiments are presented as the mean ±SD. Statistical was assessed by unpaired t-test, ns means no significance. **(D)** Wound healing analysis for assessing migration of AGS-shNC/AGS-sh1-CST1/AGS-sh2-CST1 at 0h, 24h, and 48h. Representative images (left panel) and quantification (right panel) are shown as indicated. Data from independent experiments are presented as the mean±SD. Statistical significance was assessed by an unpaired t-test. *****P*<0.0001.**(E)** Transwell migration and Matrigel invasion assays were performed to assess migration and invasion ability of CST1-knockdown AGS stable cell lines. Representative images (left panel) and quantification (righter panel) are shown as indicated. Data from independent experiments are presented as the mean±SD. Statistical significance was assessed by an unpaired t-test. *****P*<0.0001. Scale bar: 100μm.

**Figure S3. (A)** RT-qPCR detected the expression of GPX4 mRNA in HGC-27-Vector/HGC-27-CST1 and MKN45-shNC/MKN45-sh1-CST1/MKN45-sh2-CST1 cells, the experiment was repeated three times. **(B-C)** In the GEO database GSE54129 and GSE66229 data sets, the Pearson correlation line analysis showed no significant correlation between CST1 and GPX4 gene expression (the *P* values were all > 0.05). **(D)**The effect of CST1 on the ubiquitination of endogenous GPX4 in gastric cancer cells. In the MKN45-shNC/ MKN45-sh1-CST1/ MKN45-sh2-CST1 cells, immunoprecipitated GPX4, respectively, and then detected the Ub level by WB. The results showed that in the negative control group, the level of ubiquitination of GPX4 was low, whereas the level of ubiquitination of GPX4 was increased in CST1-knockdown MKN45-sh1-CST1/MKN45-sh2-CST1 cells. L.E: long exposure.

**Figure S4. (A)** The 3D spatial structures of CST1, OTUB1 and GPX4 were obtained from SWISS-MODE and PDB protein databases. **(B)** The interface area and free energy of the predicted complexes were analyzed and shown. The larger the interface area implies the easier the proteins bind to each other. Negative free energy indicates that the protein can bind stably. **(C)** “Eyelash figure” of protein-protein interactions, the protein docking regions between CST1, OTUB1 and GPX4 were shown.

**Figure S5.** Prussian blue staining, intracellular Fe^3+^ were stained as blue particles. **(A)** In HGC-27-Vector/HGC-27-CST1 cells, compared with the DMSO group, the intracellular blue particles did not change significantly after erastin treatment. **(B)** In MKN45-shNC/MKN45-sh1-CST1/MKN45-sh2-CST1 cells, compared with the DMSO group, the intracellular blue particles did not change significantly after erastin or combined with liproxstatin-1 treatment.

**Figure S6. (A)** RT-qPCR detected the expression of E-cadherin, N-cadherin and Snail mRNA in HGC-27-Vector/HGC-27-CST1 and MKN45-shNC/MKN45-sh1-CST1/MKN45-sh2-CST1 cells, the experiment was repeated three times, ***<0.001, ****<0.0001. **(B)** Gene set enrichment analysis (GSEA) showed that the gene sets related to invasiveness and degradation of the extracellular matrix were enriched in samples with high CST1 expression. **(C)** In GSE54129 and GSE66229 datasets, pearson correlation analysis revealed a significant positive correlation between CST1 mRNA and MMP9 mRNA(*P*<0.001); there was no significant correlation between CST1 and E-cadherin, Vimentin(*P*>0.05).

**Figure S7. CST1 promotes gastric cancer cell migration and invasion by regulating GPX4-K11 site ubiquitination. (A)** The online sites GPS-Uber and BDM-PUB predict potential ubiquitination sites for GPX4. **(B)** HGC-27-Vector/HGC-27-CST1 stable gastric cancer cells were transfected with GPX4-K11 site mutant, GPX4-WT plasmid, empty control plasmid and Ub-HA plasmid, respectively, and immunoprecipitated Myc tag. The ubiquitination level of the precipitated protein was detected by WB. **(C-D)** Transwell assay investigated whether GPX4-K11 ubiquitination site mutation affects the migration and invasion ability of gastric cancer cells.

**Figure S8. (A)** Western blot analysis was performed using an antibody against GPX4 in 5 pairs of GC patients’ samples. **(B)** Western blot analysis showing the expression of GPX4 in GES-1 and different GC cell lines. **(C)** Western blot analysis was performed using an antibody against CST1, GPX4 in 5 pairs of colon cancer patients’ samples. **(D)** Western blot analysis showing the expression of CST1 and GPX4 in HIEC and different colon cancer cell lines. Total GAPDH was used as a loading control.
