# Supplementary material for: CST1 inhibits ferroptosis and promotes gastric cancer metastasis by regulating GPX4 protein stability via OTUB1
Source: Oncogene. 2022 Nov 12;42(2):83–98. doi: 10.1038/s41388-022-02537-x (PMC9816059; doi:10.1038/s41388-022-02537-x)
Supplement: Supplementary file 11 — Table S2 [file 41388_2022_2537_MOESM11_ESM.docx]

**Table S2.** The correlation between clinicopathological parameters and CST1 protein expression in 95 GC

| Parameters | Cases | CST1 Expression | | | *P* Value |
| --- | --- | --- | --- | --- | --- |
|  |  | High group | Low group |  |  |
| Age (years)  <60  ≥60 | 28  67 | 16  32 | 12  35 |  | 0.404 |
| Gender  Male  Female | 72  23 | 33  15 | 39  8 |  | 0.106 |
| Tumor size (cm)  <5  ≥5 | 27  66 | 13  33 | 14  33 |  | 0.363 |
| Differentiation Moderately-well  Poorly | 23  72 | 7  41 | 16  31 |  | 0.027* |
| Depth of invasion  T1+T2  T3+T4 | 12  83 | 6  42 | 6  41 |  | 0.969 |
| Lymph node invasion  No  Yes  Distant metastasis  No  Yes | 20  75  91  4 | 6  42  47  1 | 14  33  44  3 |  | 0.039*  0.297 |
| TNM stage  I + II  III + IV | 34  61 | 16  32 | 18  29 |  | 0.614 |
