# Supplementary material for: CST1 inhibits ferroptosis and promotes gastric cancer metastasis by regulating GPX4 protein stability via OTUB1
Source: Oncogene. 2022 Nov 12;42(2):83–98. doi: 10.1038/s41388-022-02537-x (PMC9816059; doi:10.1038/s41388-022-02537-x)
Supplement: Supplementary file 12 — Table S3 [file 41388_2022_2537_MOESM12_ESM.docx]

**Table S3.** Univariate and multivariate Cox analyses of various potential prognostic factors in GC patients

|  | Univariate Cox analysis | | Multivariate Cox analysis | | | |
| --- | --- | --- | --- | --- | --- | --- |
|  | HR (95% CI) | *P* | HR (95% CI) | *P* | | |
| Age (≥60/<60) | 1.095(0.667-1.799) | 0.720 | - | | - | |
| Gender (male/female) | 0.599(0.357-1.006) | 0.053 | - | | - | |
| Differentiation  (poorly/ moderately-well) | 1.306(0.741-2.300) | 0.356 | - | | - | |
| Tumor size  (≥5cm/<5cm) | 1.363(0.810-2.295) | 0.244 | - | | - | |
| Depth of invasion (T3+T4/T1+T2)  Lymph node invasion  (Yes/No)  Distant metastasis  (Yes/No) | 3.341(1.340-8.328)  1.749(0.898-3.407)  1.025(0.372-2.823) | 0.010*  0.101  0.962 | 3.072(1.221-7.726)  -  - | | 0.017*  -  - |  |
| TNM Stage  (III-IV/I-II) | 1.763(1.057-2.940) | 0.030* | 1.512(0.903-2.534) | | 0.116 | |
| CST1 protein  (high/low) | 1.669(1.054-2.643) | 0.029* | 1.678(1.060-2.657) | | 0.027* | |

HR: hazard ratio; CI: confidence interval; **P* < 0.05.
