# Supplementary material for: CST1 inhibits ferroptosis and promotes gastric cancer metastasis by regulating GPX4 protein stability via OTUB1
Source: Oncogene. 2022 Nov 12;42(2):83–98. doi: 10.1038/s41388-022-02537-x (PMC9816059; doi:10.1038/s41388-022-02537-x)

武汉普诺赛生命科技有限公司

Procell Life Science&Technology Co,.Ltd.

HGC-27（人胃癌细胞（未分化））

Cat No.: CL-0107

1.Origin and General Characteristics

**Cell Name**	HGC-27
**Organism**	Homo sapiens, human
**Age**	
**Tissue**	
**Morphology**	epithelial
**Growth Properties**	adherent
**Descriptions**	
**Biosafety Level**	

2.Culture Conditions and Handling

**Complete Growth Medium**	RPMI-1640 (PM150110)+20% FBS (164210-500)+1% P/S (PB180120)
**Subculturing**	Remove and discard culture medium. Briefly rinse the cell layer with DPBS solution to remove all traces of serum that contains trypsin inhibitor.<br>Add 1.0 to 2.0 mL of Trypsin-EDTA solution to flask and observe cells under an inverted microscope until cell layer is dispersed (usually within 3 to 5 minutes). Cells that are difficult to detach may be placed at 37°C to facilitate dispersal.<br>Add 4.0 to 6.0 mL of complete growth medium and aspirate cells by gently pipetting. Add appropriate aliquots of the cell suspension to new culture vessels.
**Subcultivation Ratio**	1:2-1:4
**Medium Renewal**	every 2 to 3 days
**Cryopreservation**	Freeze medium: 50% basal medium+40% FBS+10% DMSO<br>Storage temperature: liquid nitrogen vapor phase
**Culture Conditions**	Atmosphere: Air, 95%; CO2, 5%<br>Temperature: 37℃

3.Special Features of the Cell Line

**Tumorigenic**	
**Effects**	
**Receptor Expression**	
**Antigen Expression**	
**Gene Expression**	
**Applications**	

Cell Line Authentication Service

HGC-27 STR Profile Report

Methodology:

Twenty short tandem repeat (STR) loci plus the gender determining locus, Amelogenin, were amplified using a commercially available STR profiling Kit. The cell line sample was processed using the ABI Prism® 3500XL Genetic Analyzer. Data were analyzed using GeneMapper® 5 software (Applied Biosystems). Appropriate positive and negative controls were run and confirmed for each sample submitted.

Data Interpretation:

Cell lines were authenticated using Short Tandem Repeat (STR) analysis as described in 2012 in ANSI Standard (ANSI/ATCC ASN-0002-2011 *Authentication of Human Cell Lines: Standardization of STR Profiling*) by the ATCC Standards Development Organization (SDO).

Procell

武汉普诺赛生命科技有限公司

Procell

Procell Life Science&Technology Co,.Ltd.

Test Results of Submitted Sample:

HGC-27 PC113				
**STR Loci**	**Allele 1**	**Allele 2**	**Allele 3**	**Allele 4**
Amelogenin	X	X		
D3S1358	17	17		
D1S1656	14	16		
D6S1043	18	18		
D13S317	10	11		
Penta E	18	18		
D16S539	7.3	10	11	
D18S51	16	17		
D2S1338	22	24		
CSF1PO	12	12		
Penta D	9	9		
TH01	9	9		
vWA	14	14		
D21S11	33	34		
D7S820	11	12	13	
D5S818	12	12		
TPOX	8	8		
D8S1179	10	11	16	
D12S391	20	21		
D19S433	14	14		
FGA	22	22		

Procell

NOTE:

Ⅰ. Loci highlighted in grey (8 core STR loci plus Amelogenin) can be made public to verify cell identity. In order to protect the identity of the donor, Please do not publish the allele calls from the STR loci tested.

Ⅱ. A relatively common occurrence of STR typing of human cancer cell lines is multiple Alleles at several loci. Three or more Alleles at one or two loci may be due to somatic mutation, trisomy or gene duplications. Events with more than three Alleles at more than three loci may be due to cellular contamination.

Ⅲ. Electropherograms showing raw data and Map are attached.

Result of searching against STR Profile Database:

Names	D5S818	D13S317	D7S820	D16S539	vWA	TH01	AM	TPOX	CSF1PO
Values	[illegible]	[illegible]	[illegible]	[illegible]	[illegible]	[illegible]	[illegible]	[illegible]	[illegible]

Options

Matches>=80% Matches>=55% Matches>=0% Another Algorithm Export Exit

Matches

NO.	Percent Match	Cell No.	Cell name	D5S818	D13S317	D7S820	D16S539	vWA	TH01	AM	TPOX	CSF1PO
			Query(Your Cell)	12,12	10,11	11,12,13	7,8,10,11	14,14	9,9	X,X	8,8	12,12
1	96%		HGC-27	12,12	10,11	11,12,13	10,11	14,14	9,9	X,X	8,8	12,12
2	96%	RCB0500	HGC-27	12,12	10,11	11,12,13	10,11	14,14	9,9	X,X	8,8	12,12

Explanation of Test Results:

Cell lines with ≥80% match are considered to be related, i.e., derived from a common ancestry. Cell lines with between a 55% to 80% match require further profiling for authentication of relatedness. Here only show cell lines with ≥80% match

STR Profile from ATCC.

Markers:

Amelogenin	X
CSF1PO	12
D3S1358	17
D5S818	12
D7S820	11,12,13
D8S1179	7,11,16 (CLS)<br>11 (PubMed=11416159; PubMed=25877200)
D13S317	10,11
D16S539	10,11
D18S51	16,17 (CLS; PubMed=25877200)<br>16 (PubMed=11416159)

Website: http://www.procell.com.cn/

Telephone: 027-87287608/82917608

E-mail: *sales@procell.com.cn*

Fax: 027-87287608

武汉普诺赛生命科技有限公司

Procell

Procell Life Science&Technology Co,.Ltd.

Addendum: Electropherogram (peak data) for Submitted Sample

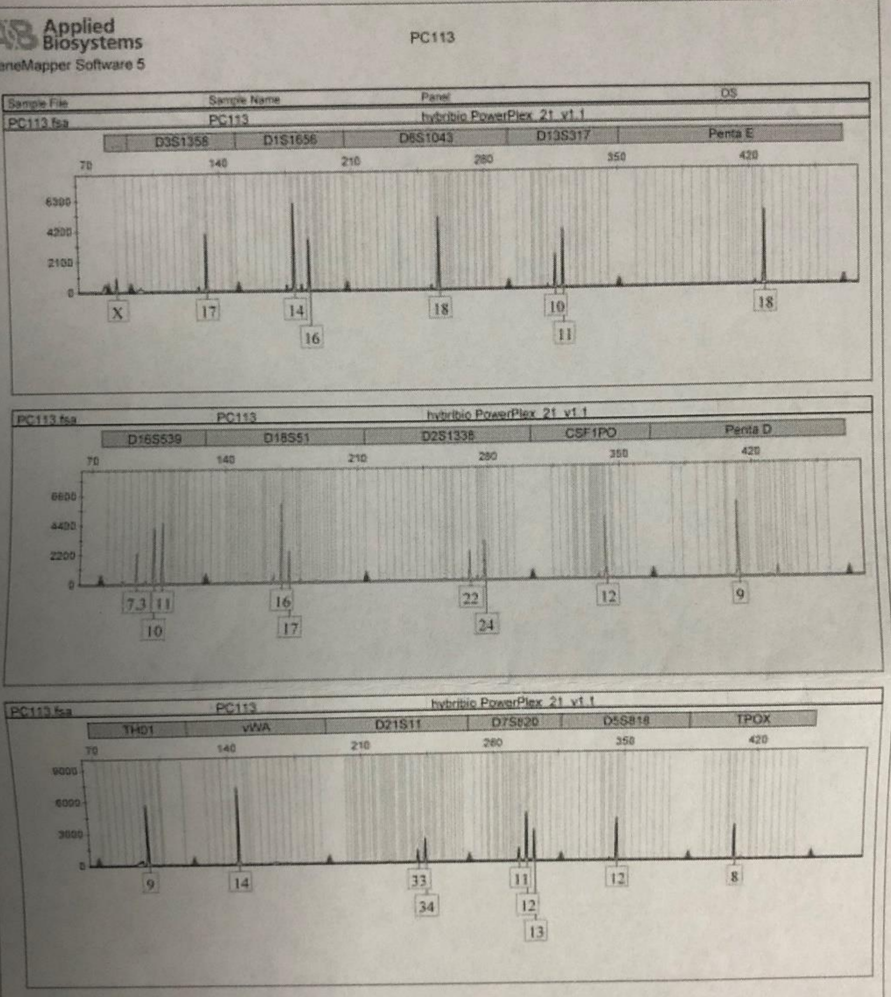

Applied Biosystems
GeneMapper Software 5

PC113

Sample File	Sample Name	Panel	OS
PC113.fsa	PC113	hybribio PowerPlex 21 v1.1	

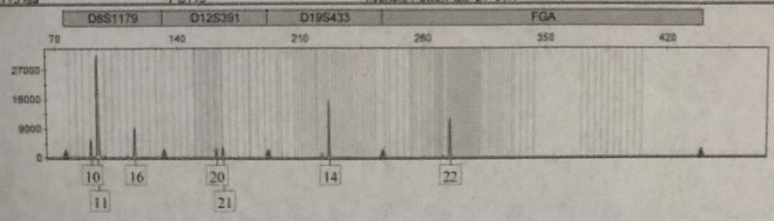

武汉普诺赛生命科技有限公司

MKN-45 细胞 STR 鉴定报告

一、 材料处理和检验方法

取适量 **MKN-45** 细胞(编号 PNS-HC-59,1×10^6)使用 Chelex100 提取 DNA，采用 21 CELLID System 扩增 20 个 STR 位点和性别鉴定位点，使用 ABI3130x1 型遗传分析仪进行 PCR 产物检测，使用 GeneMapper IDX 软件（Applied Biosystems）对检测结果进行分析，并与 ATCC、DSMZ、JCRB、Cellosaurus 等数据库进行比对。

二、 检测结果

实验中阴性及阳性对照结果均正确。

MKN-45 细胞株的 STR 位点和 Amelogenin 位点的基因分型结果见附表，分型图谱见附图。

三、 分析说明

MKN-45 细胞株基因组 DNA 扩增后图谱清晰，分型结果良好。

四、 检验结论

1. **MKN-45** 细胞株 DNA 进行细胞 STR 分型结果显示，细胞株中未发现人类细胞交叉污染。
2. 该细胞株 DNA 分型在细胞库中找到与其细胞分型 100.00%相匹配的细胞株，细胞株名称为 **MKN-45**。

www.procell.com.cn Procell

附表 1：细胞株 **MKN-45** 的 STR 位点和 Amelogenin 位点的基因分型结果

STR Loci	样品名称：PNS-HC-59	数据库名称：MKN-45
Amelogenin	X	X
CSF1PO	12	12
D2S1338	18	
D3S1358	15,16	15,16
D5S818	10,11	10,11
D7S820	10,11	10,11
D8S1179	13,17	13,17
D13S317	8,11	8,11
D16S539	10	10
D18S51	16	16
D19S433	14,16.2	14,16.2
D21S11	31	31
FGA	19,24	19,24
PentaD	10	10
PentaE	10	10
TH01	7	7
TPOX	8	8
vWA	19	19
D1S1656		
D6S1043	14	
D12S391	26	
D2S441	11	
Cellosaurus 数据库匹配度 100.00%，匹配位点数 16（https://web.expasy.org/cellosaurus-str-search/）		

www.procell.com.cn

备注：

1. 根据国际细胞鉴定委员会(ICLAC)制定的细胞 STR 鉴定标准，细胞系的匹配度≥80%时，认为它们具有相关性，即衍生于共同的祖先细胞；匹配度在 55% 至 80% 之间，需要进一步验证相关性；小于 55%，表明两者不具有相关性。
2. 图谱有效峰为真实的 PCR 条带;小峰和非特异性条带在计算中忽略不计。
3. STR 数据比对结果默认 ExPASy，数据来源包括 ATCC，DSMZ，JCRB 等细胞库以及文献和资料记载，数据库入口 https://web.expasy.org/cellosaurus-str-search/。

附图 1：MKN-45 细胞 STR 位点和 Amelogenin 位点的基因分型结果

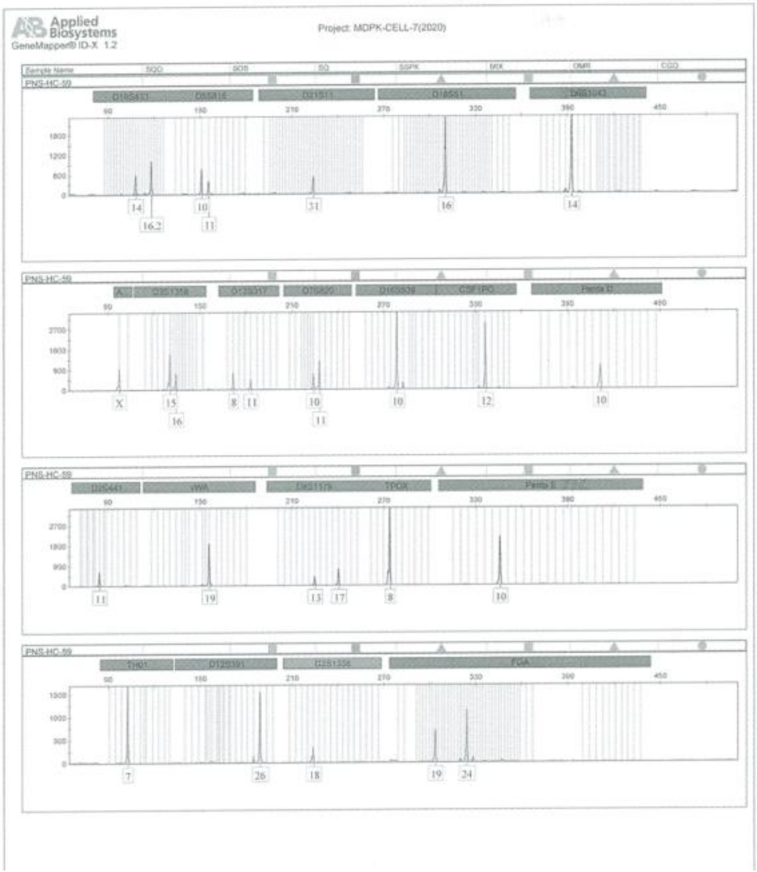

Supplement: Supplementary file 13 — Supplementary file 1-Cell Line STR Identification Reports [file 41388_2022_2537_MOESM13_ESM.pdf]
